# Interferon regulatory factor 1‐triggered free ubiquitin protects the intestines against radiation‐induced injury via CXCR4/FGF2 signaling

**DOI:** 10.1002/mco2.168

**Published:** 2022-08-26

**Authors:** Yang Jiao, Jing Xu, Bin Song, Ailing Wu, Lu Pan, Ying Xu, Fenghao Geng, Xiaoqian Li, Congzhao Zhao, Min Hong, Xuanyu Meng, Judong Luo, Pengfei Liu, Ming Li, Wei Zhu, Jianping Cao, Shuyu Zhang

**Affiliations:** ^1^ School of Radiation Medicine and Protection Medical College of Soochow University Suzhou China; ^2^ State Key Laboratory of Radiation Medicine and Protection and Collaborative Innovation Center of Radiation Medicine of Jiangsu Higher Education Institutions Soochow University Suzhou China; ^3^ Laboratory of Radiation Medicine West China Second University Hospital Sichuan University Chengdu China; ^4^ Second Affiliated Hospital of Chengdu Medical College China National Nuclear Corporation 416 Hospital Chengdu China; ^5^ West China School of Basic Medical Sciences & Forensic Medicine Sichuan University Chengdu China; ^6^ Department of Oncology The Affiliated Changzhou No. 2 People's Hospital of Nanjing Medical University Changzhou China; ^7^ NHC Key Laboratory of Nuclear Technology Medical Transformation, Mianyang Central Hospital Mianyang China

**Keywords:** fibroblast growth factor‐2, free ubiquitin, ionizing radiation, radiation‐induced intestinal injury

## Abstract

Radiation‐induced intestinal injury is a serious concern during abdominal and pelvic cancers radiotherapy. Ubiquitin (Ub) is a highly conserved protein found in all eukaryotic cells. This study aims to explore the role and mechanism of free Ub against radiogenic intestinal injury. We found that free Ub levels of irradiated animals and human patients receiving radiotherapy were upregulated. Radiation‐induced Ub expression was associated with the activation of interferon regulatory factor 1 (IRF1). Intraperitoneal injection of free Ub significantly reduced the mortality of mice following 5–9 Gy total body irradiation (TBI) through the Akt pathway. Free Ub facilitates small intestinal regeneration induced by TBI or abdominal irradiation. At the cellular level, free Ub or its mutants significantly alleviated cell death and enhanced the survival of irradiated intestinal epithelial cells. The radioprotective role of free Ub depends on its receptor CXCR4. Mechanistically, free Ub increased fibroblast growth factor‐2 (FGF2) secretion and consequently activated FGFR1 signaling following radiation in vivo and in vivo. Thus, free Ub confers protection against radiation‐induced intestinal injury through CXCR4/Akt/FGF2 axis, which provides a novel therapeutic option.

## Graphical abstract

IRF1 is activated by ionizing radiation and promotes ubiquitin (Ub) expression. Free Ub activates the cellular membrane receptor CXCR4, which increases Akt phosphorylation and FGF2 secretion. FGF2 protects irradiated intestinal cells through its receptor FGFR1. Thus, free Ub confers protection against radiation‐induced intestinal injury through the CXCR4/FGF2 axis.

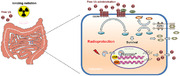

## 1 INTRODUCTION

Radiation facilities and radioactive materials have been widely used in military, industry, medicine, science, and nuclear facilities, which have significantly increased the potential of large‐scale, uncontrolled exposure to radiation.^1^, ^2^ The gastrointestinal tract, especially the small intestine, is particularly sensitive to radiation, rendering it vulnerable to the effects of collateral radiation from the abdominal and pelvic cancers radiotherapy.^2^, ^3^, ^4^ Moreover, radioactive materials have been widely implemented in industrial, medicinal, and scientific applications as well as in military and nuclear facilities, which have significantly increased the potential of the large‐scale, uncontrolled public exposure to radiation. Radiation accidents, such as those occurred in Three Mile Island nuclear power station, United States (1979); Chernobyl, Russia (1988); and Fukushima, Japan (2011), have highlighted the potential threats associated with catastrophic nuclear events,^2^, ^5^ which primarily manifest as injury to the hematologic, gastrointestinal, dermatologic, and central nervous systems. However, radiation‐induced injury is a complex pathophysiological process, which remains a clinical challenge to practicing physicians, due to the deficiency of accurate diagnosis and specific therapeutic regimens. Therefore, radiological threat remains exacerbated by the lack of available countermeasures.^6^

Ubiquitin (Ub) is a highly conserved protein with 76 amino acid residues universally expressed in all eukaryotic cells. The Ub molecules are encoded by four different genes: *UBA52*, *RPS27A*, *ubiquitin B* (*UBB*), and *ubiquitin C* (*UBC*). Individual Ub is processed from their primary polyubiquitin precursor proteins.^7^, ^8^ The covalent linkage of Ub to a variety of cellular proteins (ubiquitination) is one of the most common posttranslational modifications in eukaryotic cells, leading to versatile signaling outcomes.^7^, ^8^ Meanwhile, Ub is a normal constituent of extracellular space and bodily fluids, which is designated as free Ub or extracellular Ub.^8^ Elevated levels of free Ub have been found in blunt trauma,^9^ burns,^10^ alcoholic liver disease,^11^ type 2 diabetes,^12^ and esophageal cancer.^13^ The functional importance of free Ub has been uncovered in limited pathological and physiological processes. Ub can mediate the growth suppression and apoptosis of hematopoietic cells^14^ and inhibit cytotoxic activity of platelets.^15^ Free Ub is found in surgical wound fluid, which provides a chemotactic signal for myeloid dendritic cells.^16^ Free extracellular Ub inhibits β‐AR‐stimulated apoptosis in adult cardiomyocytes.^17^ CXC chemokine receptor 4(CXCR4, CD184) has been identified as a natural receptor for free Ub.^18^ Ub and stromal cell‐derived factor‐1α (SDF‐1α) function through distinct receptor interactions.^19^ In 2006, Romanenko et al. reported that Ub levels were significantly upregulated in tissue samples of 45 patients with urinary system diseases who lived in ^137^Cs‐contaminated areas for a long time after the Chernobyl accident.^20^ We previously reported that Ub was highly expressed in lung cancer tissues and that the knockdown of Ub coding genes sensitized lung cancer cells to radiation,^21^ suggesting that Ub is likely associated with tissue radiosensitivity.

However, there are no evidence‐based studies on the radioprotective effects of free Ub. To explore the biological functions of free Ub against radiogenic disorders, we investigated the Ub level in tissues and serum following radiation and the radioprotective role of Ub. Our results showed that free Ub administration significantly boosted the overall survival of mice exposed to radiation meanwhile ameliorated radiation‐induced intestinal mucosal injury through CXCR4/FGF2/Akt axis.

## 2 RESULTS

### 2.1 Ionizing radiation modulates free ubiquitin expression and release

**FIGURE 1:**
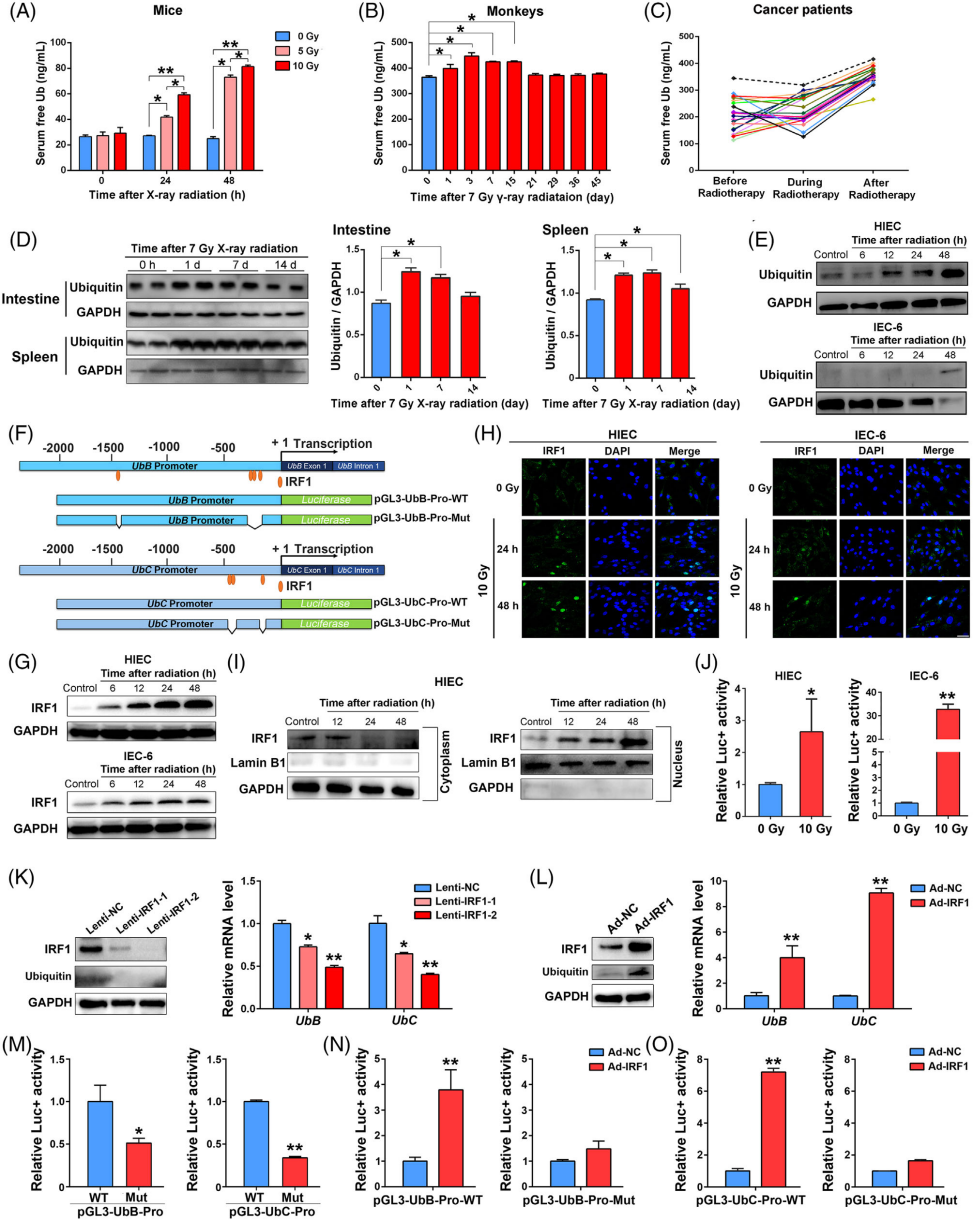
Free ubiquitin is induced by ionizing radiation, which is regulated by Interferon regulatory factor 1 (IRF1). (A) Serum from nonirradiated control mice and irradiated mice was collected and assayed for free ubiquitin (Ub) by ELISA (*n* = 3). (B) Free ubiquitin levels of monkey serum at different time points after radiation (*n* = 6). (C) Free ubiquitin levels of human patients receiving radiotherapy (*n* = 20, including 11 esophageal cancer patients, 4 lung cancer patients and 5 rectal cancer patients). (D) Western blotting analyses of ubiquitin expression in mouse intestines and spleens after radiation. (E) Western blotting analyses of ubiquitin expression in intestinal cells exposed to radiation. (F) Schematic map of human *UbB* and *UbC* promoter. Bioinformatics analysis predicted several IRF1 binding sites in the proximal promoter of *UbB* and *UbC*. The promoter region of the genes was cloned upstream of a luciferase reporter gene. (G) Western blotting analyses of IRF1 expression at different timepoints post radiation in human intestinal epithelial cell (HIEC) and intestinal epithelial cell (IEC)‐6 cells. (H) Immunofluorescence assay of IRF1 distribution. Scale bar = 20 μm. (I) Western blotting analysis of cytoplasmic and nuclear IRF1. (J) Relative luciferase activity of IRF1‐repsonsive reporter with 0 or 10 Gy radiation. (K) The effect of IRF1 silencing on *UbB* and *UbC* mRNA levels. (L) The effect of IRF1 overexpression on *UbB* and *UbC* mRNA levels. (M) Deletion of predicted IRF1 binding sites reduced promoter activity in HIEC cells. The effect of IRF1 on the luciferase reporter activity harboring (N) *UbB* and (O) *UbC* promoters. Data are presented as mean ± SEM. *P < 0.05; ** *P* < 0.01 compared with the control group

Increased Ub levels have been reported during the progression of several diseases.^9^, ^10^, ^11^, ^12^, ^13^ To investigate serum‐free Ub levels in response to radiation in vivo, C57BL/6J mice were first exposed to 0, 5, and 10 Gy X‐rays. Increased serum‐free Ub concentrations were observed in both dose‐dependent and time‐dependent manners using ELISA (Figure 1A). As shown in Figure 1B, radiation‐induced serum‐free Ub enhancement was observed in the radiation‐induced injury model of rhesus monkey (*Macaca mulatta*), 15 days immediately after 7 Gy  γ‐ray exposure using a ^60^Co source compared to non‐irradiated control (*P* < 0.01). Additionally, obvious increases in serum‐free Ub were also confirmed in 20 cancer patients including rectal cancer patients receiving radiotherapy (*P* < 0.05; Figure 1C). The current study suggested that the increased serum level of free Ub is related to radiation exposure.

We then characterized free Ub expression in mouse tissues following ionizing radiation. Using Western blotting analysis, we found that Ub protein was radio‐induced in mouse intestine and spleen tissues but not in the lung, kidney, liver, and heart tissues (Figure 1D and Figure S1). The mRNA level of Ub coding gene *UbB* but not *UbC* was increased in irradiated mouse intestine (Figure S2). To determine free Ub expression at the cellular level following radiation, intestinal epithelial cell lines (IEC)‐6 and human intestinal epithelial cells (HIEC) were exposed to radiation. Results showed that ionizing radiation obviously induced free Ub expression (Figure 1E). Taken together, these results demonstrated that free Ub expression was induced by ionizing radiation in vitro and in vivo.

### 2.2 Free ubiquitin expression is positively regulated by interferon regulatory factor 1 following radiation

Since Ub was inducible by ionizing radiation, we next investigated upstream factor(s) driving Ub expression. Bioinformatics analysis predicted several putative IRF1 binding sites in the proximal promoter of the two Ub coding genes named *UbB* and *UbC* (Figure 1F). Since IRF1 has been reported to affect diverse physiological and pathological events including stress response,^22^, ^23^ the role of this transcriptional factor in modulating Ub was further characterized. Ionizing radiation upregulated IRF1 protein levels in a time‐dependent manner (Figure 1G) and promoted the nuclear translocation of IRF1(Figure 1H,I). Moreover, IRF1‐responsive reporter was significantly activated after radiation exposure (Figure 1J). Silencing of IRF1 decreased the mRNA levels of both *UbB* and *UbC* (Figure 1K), whereas forced expression of IRF1 significantly upregulated *UbB* and *UbC* transcription (Figure 1L), indicating that IRF1 positively regulated Ub transcription. Luciferase vectors containing wild‐type or IRF1‐binding sites deleted promoters of *UbB* and *UbC* were constructed (Figure 1G) and each transfected into intestinal cells. Ablation of the predicted IRF1 binding sites significantly compromised *UbB* and *UbC* promoter activity, compared with their respective wild‐type promoter (Figure 1M). Overexpression of IRF1 by an adenovirus significantly increased the luciferase activity with wild‐type *UbB* and *UbC* promoters (Figure 1N,O). However, IRF1‐mediated activation was not observed when cells were transfected with the promoter reporters without IRF1 binding sites (Figure 1G,N,O). Moreover, in *IRF1^–/–^* intestinal tissues, Ub levels were lower than that from *IRF1^+/–^* mice (Figure S3). Taken together, these results clearly indicated that IRF1 was activated by ionizing radiation and positively regulated Ub expression.

### 2.3 Free ubiquitin improves mouse survival and tissue injuries after irradiation

**FIGURE 2:**
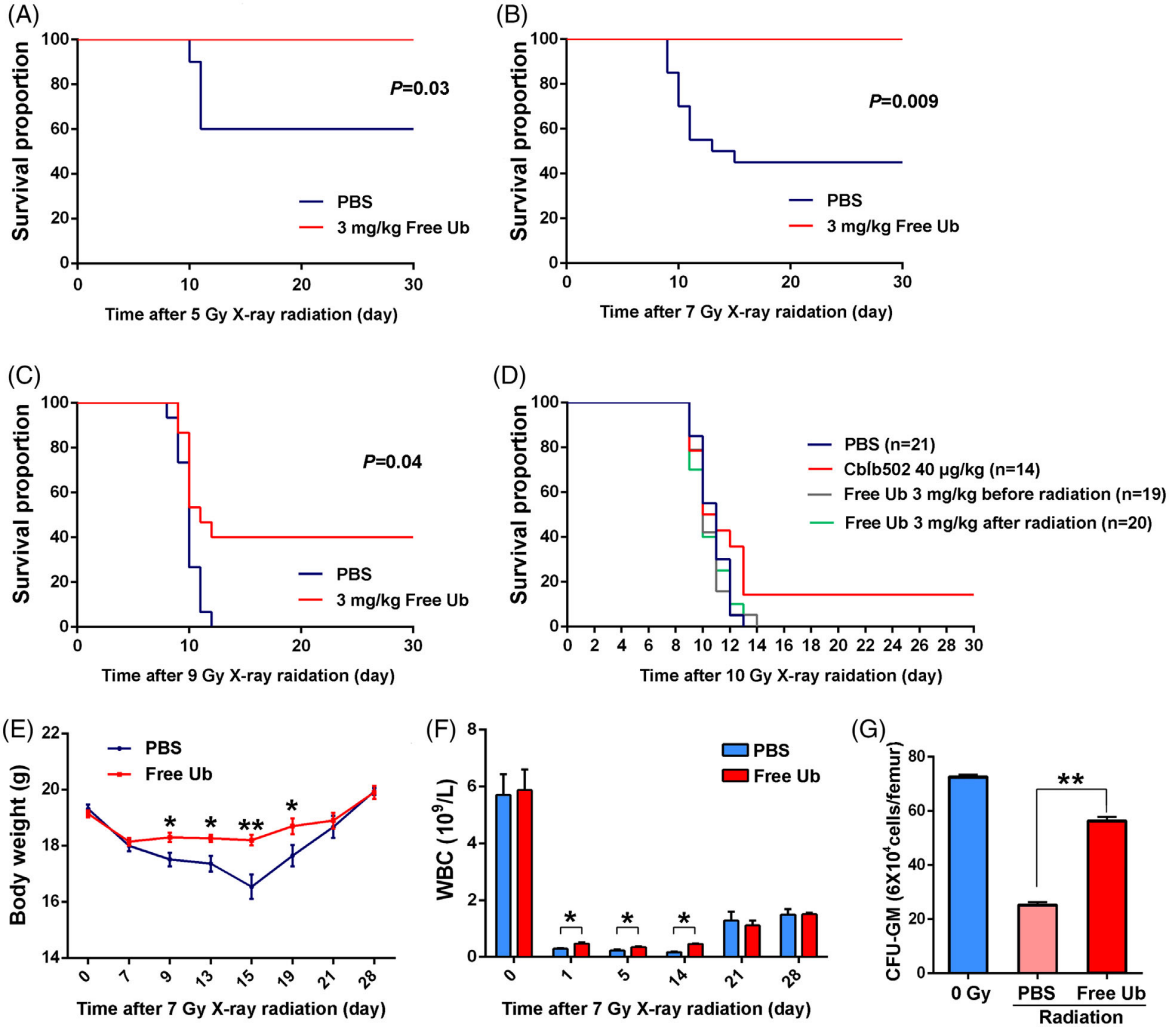
Free ubiquitin improves the survival of mice after lethal doses of radiation. (A) Kaplan–Meier survival analyses of C57BL/6J mice after 5 Gy total body irradiation (TBI) treated with PBS or free ubiquitin (Ub). *n* = 20 for each group. (B) Kaplan–Meier survival analyses of C57BL/6J mice after 7 Gy TBI treated with PBS or free Ub. *n* = 20 for each group. (C) Kaplan–Meier survival analyses of C57BL/6J mice after 9 Gy TBI treated with PBS or Ub. *n* = 20 for each group. (D) Kaplan–Meier survival analyses of C57BL/6J mice after 10 Gy TBI treated with PBS, Cblb502 or free Ub. (E) C57BL/6J mice were intraperitoneally injected with PBS or free Ub followed by 7 Gy TBI. Body weight change of each group of mice. (F) White blood cells (WBCs) in mice of different groups after 7 Gy TBI. (G) The number of colony forming units of granulocyte macrophage (CFU‐GM) in each group. *P < 0.05; **P < 0.01 compared with the control group

To investigate its effects on irradiated mouse survival, recombinant free Ub was verified by Coomassie staining and Western blotting (Figure S4), and then administered i.p. to C57BL/6J mice in different concentrations followed by total body irradiation (TBI), which has well accepted as a gastrointestinal injury mode.^24^ The animal survival was monitored for up to 30 days, and the results showed that 3 mg/kg free Ub exhibited the best radioprotection (Figure S5). TBI at 5 and 7 Gy caused 40%–50% lethality in phosphate buffered saline (PBS)‐treated C57BL/6J mice within 30 days, whereas the free Ub treatment significantly improved the survival rate to 100% (*P* < 0.05, Figure 2A,B). Free Ub also extended survival time after 9 Gy TBI, when compared with the control group (*P* = 0.04, Figure 2C). However, free Ub could not reduce the mortality of mice when exposed to 10 Gy radiation (Figure 2D), which was inferior to radioprotective Cblb502^25^ at this dose. Moreover, di‐Ub did not show the radioprotective effect (Figure S6). These data revealed the novel finding that free Ub significantly increased the overall survival of mice exposed to lethal irradiation.

We next explored whether free Ub mitigated radiogenic tissue injuries. Compared with the PBS‐treated group, free Ub administration mitigated 7 Gy radiation‐induced weight loss in mice (Figure 2E). In mice exposed to 7 Gy TBI, significantly impaired peripheral white blood cells (WBC) and eliminated colony forming units of granulocyte macrophage (CFU‐GM) were observed, as compared to the control group (Figure 2F,G). However, the number of WBC in free Ub‐injected mice increased significantly following radiation at days 1, 5, and 14 in comparison to the PBS‐treated mice (Figure 2F). And the CFU‐GM number was restored by free Ub treatment, which was almost equivalent to the sham radiation control group at day 7 post 7 Gy TBI, as compared to the PBS‐injected group (Figure 2G). These results indicated that free Ub can protect multiple organs and tissues from radiation‐induced damage.

### 2.4 Free ubiquitin attenuated radiogenic intestinal injury through activation of Akt

**FIGURE 3:**
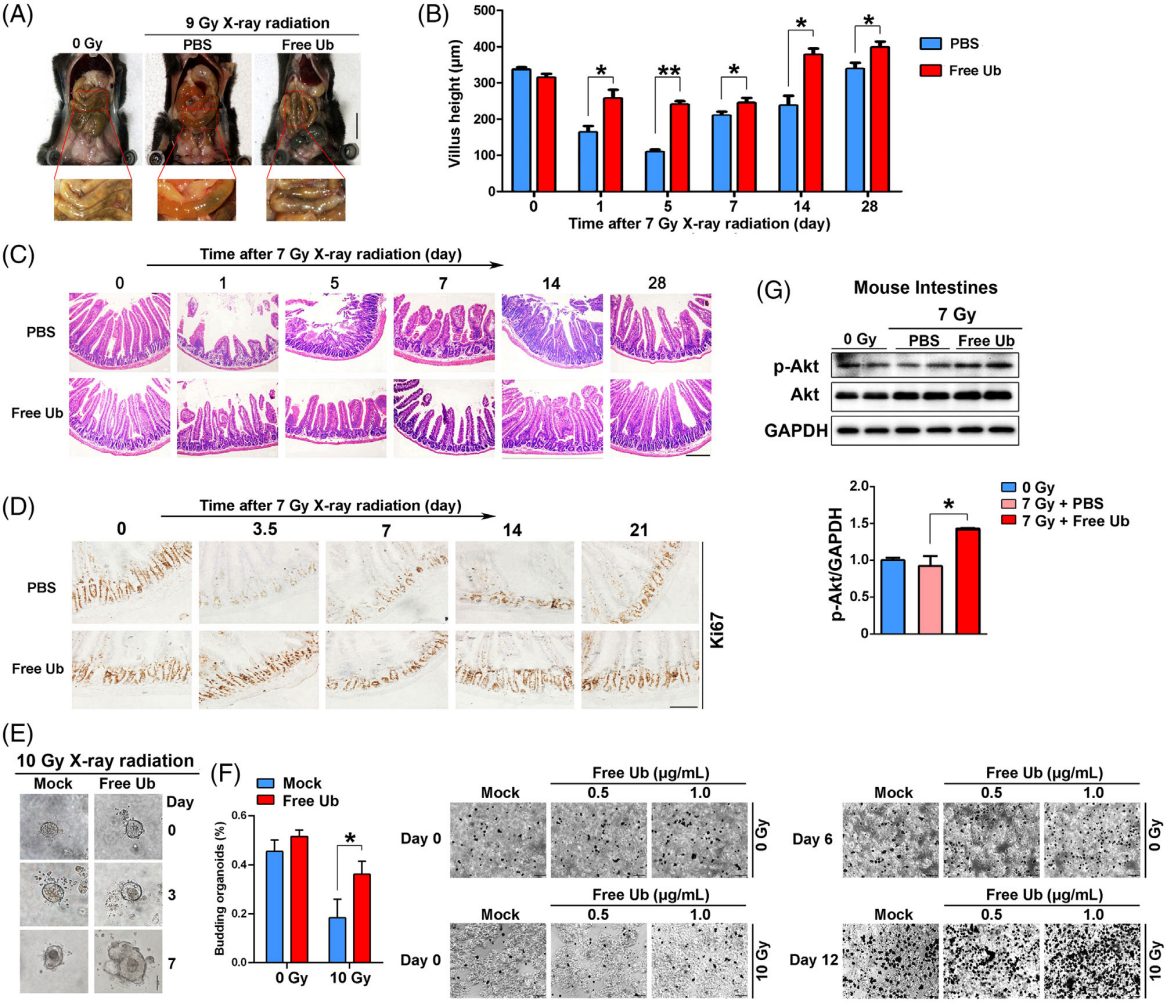
Free ubiquitin attenuates radiation‐induced intestinal damage. (A) Representative images of mouse intestines from PBS or free ubiquitin (Ub)‐treated mice at 3.5 days after total body irradiation (TBI) (*n* = 3). Scale bar = 20 μm. (B) Villus height and crypt depth were determined by calculating H&E‐stained sections (*n* = 4). The images were analyzed using Image J software. At least 30 well‐oriented, full‐length crypt‐villus units per mouse were measured. (C) Representative H&E staining of mouse intestine from PBS or free Ub‐treated mice after 7 Gy TBI. Scale bar = 100 μm. (D) Representative Ki67‐immunostained intestinal sections from PBS or free Ub‐treated mice at different time points after 7 Gy TBI. Scale bar = 50 μm. (E) Representative phase contrast images of an intestinal organoid cultured with or without free Ub (0.5 μg/ml) following radiation. Scale bar = 50 μm. (F) Representative phase contrast images and calculated budding percentage of intestinal organoids (*n* = 5). Black dots represent organoids. Scale bar = 200 μm. (G) The expression of Akt and p‐Akt in mouse intestines of indicated groups (*n* = 4). *P < 0.05; **P < 0.01 compared with the control group

To examine the putative role of free Ub against radiation‐induced intestinal injury, we compared anatomical, histological, and molecular manifestations within mouse intestines treated with PBS or free Ub post TBI. As shown in Figure 3A, free Ub attenuated radiation‐induced intestinal ulceration and edema of mice. Results from H&E showed that free Ub well preserved the crypt‐villus structure in the small intestines and significantly increased villus height at days 1, 5, and 14 post‐TBI (Figure 3B,C). Moreover, to simulate radiation‐induced intestinal injury during cancer radiotherapy, and to exclude hematopoietic injury caused by TBI,^24^ mice exposed to 20 Gy abdominal irradiation were applied to confirm the radioprotection effect of free Ub for small intestine (Figure S7 and Figure 6C). Consistently, the positive staining of the cell‐cycle state marker Ki67 in mouse intestine was significantly higher in free Ub‐treated mice following radiation exposure, particularly at 3.5 days, than in PBS‐treated irradiated controls (Figure 3D). The radioprotective role of free Ub was further validated in an ex vivo intestinal organoid culture system (Figure 3E). The crypts treated with free Ub showed significantly increased organoid‐formation capacity than those from sham‐irradiated crypts (Figure 3F). These results indicated that free Ub attenuated radiation‐induced intestinal injury.

Since free Ub has been shown to activate the phosphorylation of Akt, we next investigated p‐Akt expression in mouse intestines treated with free Ub or PBS. Results showed that radiation reduced the level of p‐Akt, which was restituted by free Ub (Figure 3G). Immunohistochemical staining (IHC) staining confirmed the increase of p‐Akt in free Ub‐treated mouse intestines (Figure 5B).

### 2.5 Free ubiquitin protects intestinal cells but not cancer cells against radiation

**FIGURE 4:**
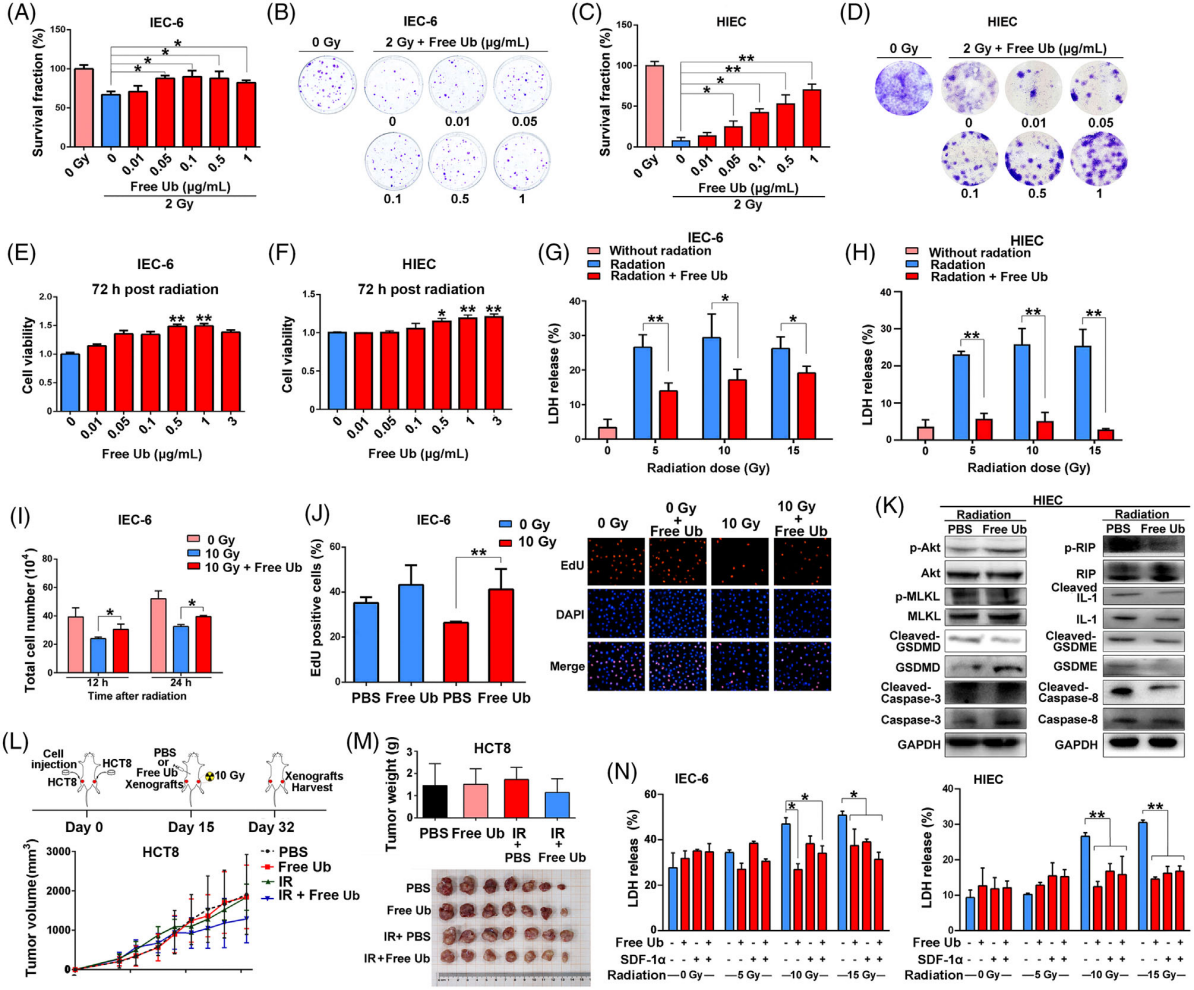
Free ubiquitin alleviates radiation‐induced cell death in intestinal epithelial cells. (A) Clonogenic cell survival assay was generated for IEC‐6 cells that were treated with indicated concentration of free ubiquitin (Ub) for 24 h and then exposed to 2 Gy radiation. The survival fraction was normalized to the unirradiated control group. (B) Representative clones from each group after 0 or 2 Gy radiation. (C) Clonogenic cell survival of human intestinal epithelial cell (HIEC) treated with indicated concentration of free Ub for 24 h and then exposed to 2 Gy radiation. (D) Representative clones from each group after 0 or 2 Gy radiation. The nonirradiated cells are more evenly distributed than irradiated cells. (E,F) IEC‐6 and HIEC cells were pretreated with indicated concentration of free Ub. Cell viability was measured 72 h after 10 Gy radiation by CCK‐8 based assay. (G,H) After incubation of free Ub for 24 h, cells were irradiated and LDH of each group was measured. (I) IEC‐6 cells were pretreated with indicated concentration of free Ub for 24 h followed by 0 or 10 Gy radiation. Relative cell number of each group was measured by a handheld cell counter (Scepter automated cell counter; Millipore, Billerica, Mexico). (J) Cell proliferation was measured in an EdU incorporation assay at 48 h after radiation. (K) Western blotting analysis of cell death related protein in HIEC cells. (L) Free Ub does not affect the radiosensitivity of xenograft tumors. HCT8 tumor bearing mice (*n* = 8) were treated with PBS or free Ub (i.p.) followed by 0 or 10 Gy radiation (IR). Tumor volumes were measured for 30 days. (M) Tumor weight and xenograft images of each group on day 30. (N) The effect of free Ub (0.5 μg/ml) plus SDF‐1α (100 ng/ml) on the LDH release of IEC‐6 and HIEC cells. After incubation of for 8 h, cells were irradiated and LDH of each group was measured 24 h after radiation. The data are presented as the mean ± SEM and normalized to the control cells. *P < 0.05; **P < 0.01 compared with the group with radiation alone

To determine the radioprotective effect of free Ub on intestinal cells, IEC‐6 and HIEC cells were first treated with different concentrations of free Ub, and up to 3.00 μg/ml free Ub treatment did not show cellular toxicity (Figure S8). The effect of free Ub on the radiosensitivity and survival of IEC‐6 cells was determined via the clonogenic survival assay. As shown in Figure 4AB, pretreatment with 0.05–1.00 μg/ml free Ub significantly increased the survival rate in IEC‐6 cells as compared with the control cells treated with irradiation alone. Similar results were obtained for HIEC (Figure 4C,D), indicating radioprotection. In addition, IEC‐6 and HIEC cells pretreated with 0.50 and 1.00 μg/ml free Ub showed a significant increase of cell viability after radiation, compared with the mock treatment (Figure 4E,F). Based on the above results, 0.50 μg/ml free Ub pretreatment was chosen for the following cellular experiments. To further evaluate the radioprotective effect of free Ub, the extracellular lactate dehydrogenase (LDH) release was detected, which was pre‐treated with or without free Ub for 24 h prior to exposure to different doses of X‐rays. As shown in Figure 4G,H, free Ub pretreatment effectively reduced LDH release in IEC‐6 and HIEC cells following 5, 10, and 15 Gy X‐ray radiation (*P* < 0.05). Compared with 10 Gy irradiated IEC‐6 cells, free Ub pretreatment significantly increased cell proliferation as evidenced by the cell number (Figure 4I) and EdU‐positive cell percentage (Figure 4J). However, free Ub pretreatment did not improve apoptotic percentage of IEC‐6 cells after exposure to X‐ray radiation (data not shown). Western blotting analysis showed that free Ub inhibited the expression of pyroptosis‐related proteins (including cleaved GSDMD, cleaved GSDME, cleaved Caspase‐8, and cleaved IL‐1), but not necroptosis and apoptosis related proteins, indicating that free Ub suppressed cell pyroptosis induced by ionizing radiation (Figure 4K).

The effect of free Ub on the radiosensitivity of colon cancer cells was further evaluated, due to the clinical relevance of the radiation therapy for its treatment. The hind limbs of nude mice were grafted with colon cancer cell line HCT8 via a subcutaneous injection. On day 15, mice were i.p. injected with PBS or free Ub, followed by 0 or 10 Gy irradiated (Figure 4L). The results showed that free Ub administration did not show the significant effect on the growth of HCT8 xenografts with or without radiation (Figure 4L,M ). These results indicated that a single administration of free Ub did not affect the growth and radiosensitivity of colon cancer cells in vivo.

Since SDF‐1α has been reported as a major ligand for CXCR4,^17^, ^18^ we therefore investigate the role of SDF‐1α in free Ub‐mediated radioprotection. To this end, IEC‐6 and HIEC cells were treated with free Ub alone or together with SDF‐1α, followed by ionizing radiation. Although SDF‐1α alone did not affect cell viability, it did decrease the LDH release in intestinal epithelial cells with more pronounced effect in HIEC cells (Figure S9 and Figure 4N). The combination of SDF‐1α with free Ub did not show more favorable protection in cell viability and cell death induced by 10 and 15 Gy radiation (Figure S9 and 4N), suggesting that SDF‐1α did not cooperate with free Ub in radioprotection.

### 2.6 The radioprotective role of free ubiquitin depends on CXCR4

**FIGURE 5:**
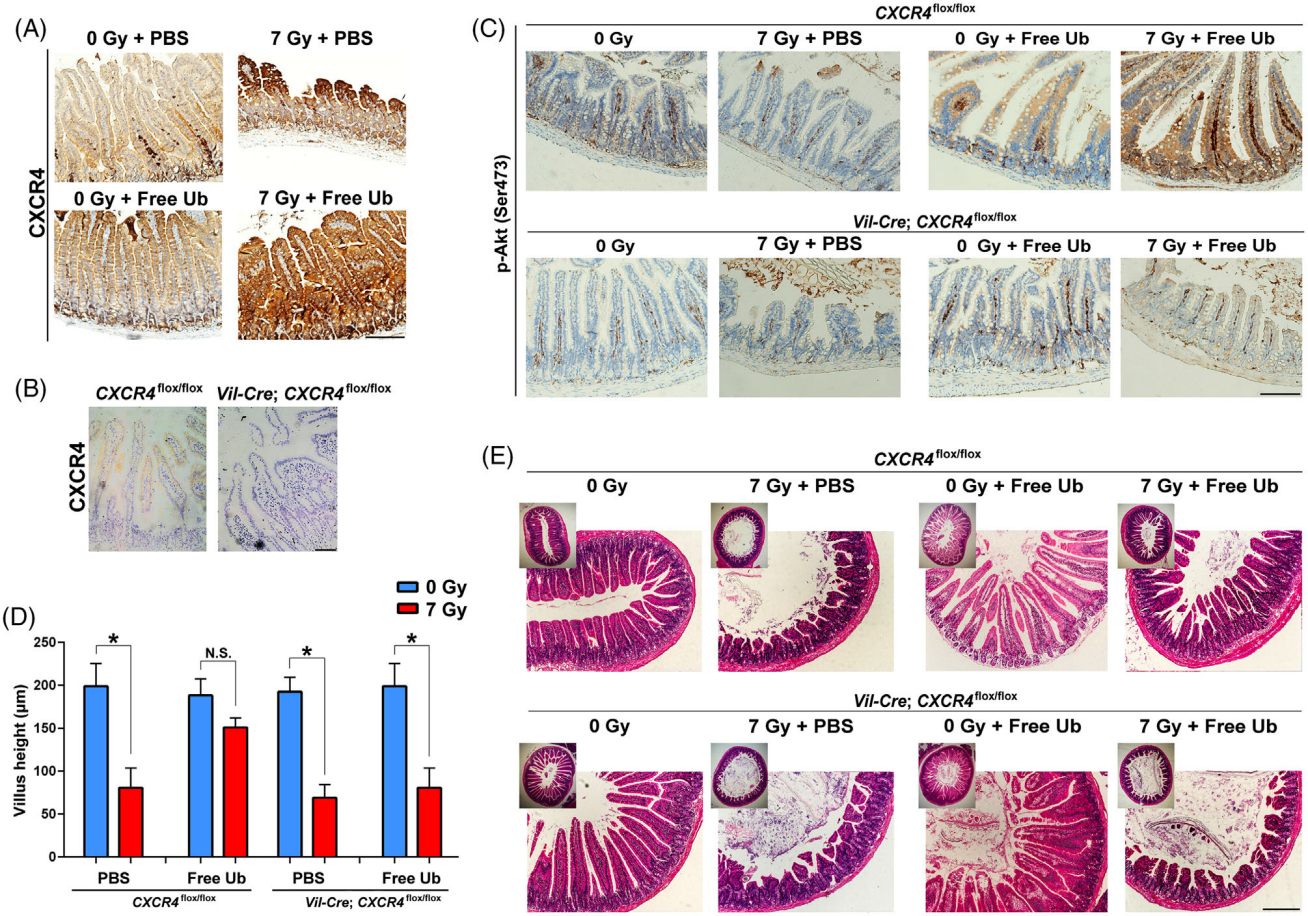
CXCR4 is involved in the radioprotective effect of free ubiquitin. (A) Mice were i.p. treated with PBS or free ubiquitin, followed by 0 or 7 Gy radiation. CXCR4 expression was measured by IHC analysis. (B) CXCR4 expression in the intestine of *Vil‐Cre*; *CXCR4* ^flox/flox^ and control mice (*CXCR4* ^flox/flox^) was confirmed by IHC (400 ×). Scale bar = 100 μm. (C) Representative images of IHC staining of Akt phosphorylation (Ser473) in intestinal tissues from *Vil‐Cre*; *CXCR4* ^flox/flox^ and control mice at 3.5 days after 7 Gy TBI. Scale bar = 100 μm. (D) Villus height was determined by taking pictures of H&E‐stained sections from *Vil‐Cre*; *CXCR4* ^flox/flox^ and control mice at 3.5 days after 7 Gy total body irradiation (TBI). The images were analyzed using Image J. At least 30 well‐oriented, full‐length crypt‐villus units per mouse were measured. Crypts per circumference were counted from three separate tubular intestinal H&E‐stained slices for each mouse. (*n* = 4). (E) Representative H&E staining images of intestinal tissues from each group. Scale bar = 100 μm

We next sought out to ascertain the expression of CXCR4, a recognized receptor for free Ub,^17^ after free Ub administration. Indeed, FITC‐labeled free Ub could not be incorporated by intestinal cells (data not shown). As shown in Figure 5A, free Ub increased the expression of CXCR4. In irradiated intestinal tissues, higher CXCR4 expression may partially attributed to increased free Ub levels. To examine the association between the free Ub /CXCR4 axis and radiogenic injury repair, we generated mice with specific knocked out *CXCR4* in the intestine. Ablation of CXCR4 expression in intestinal tissues was confirmed by IHC (Figure 5B). *Vil‐CXCR4^flox^* ^/^ *^flox^*, and control mice were irradiated with 7 Gy X‐ray. IHC staining showed that the p‐Akt in free Ub‐treated mouse intestines was compromised in *CXCR4* knockout mice, suggesting that CXCR4 mediated Akt activation by free Ub (Figure 5C). As shown in Figure 5D,E, intestinal injury could be mitigated by free Ub not in the intestinal‐specific *CXCR4* knockout mice (*Vil‐Cre; CXCR4^flox^* ^/^ *^flox^*), but in the control mice, as evidenced by disrupted villi by radiation. These results indicated that the radioprotective role of free Ub depends on CXCR4.

### 2.7 The effect of ubiquitin mutants on radiation‐induced intestinal injury

**FIGURE 6:**
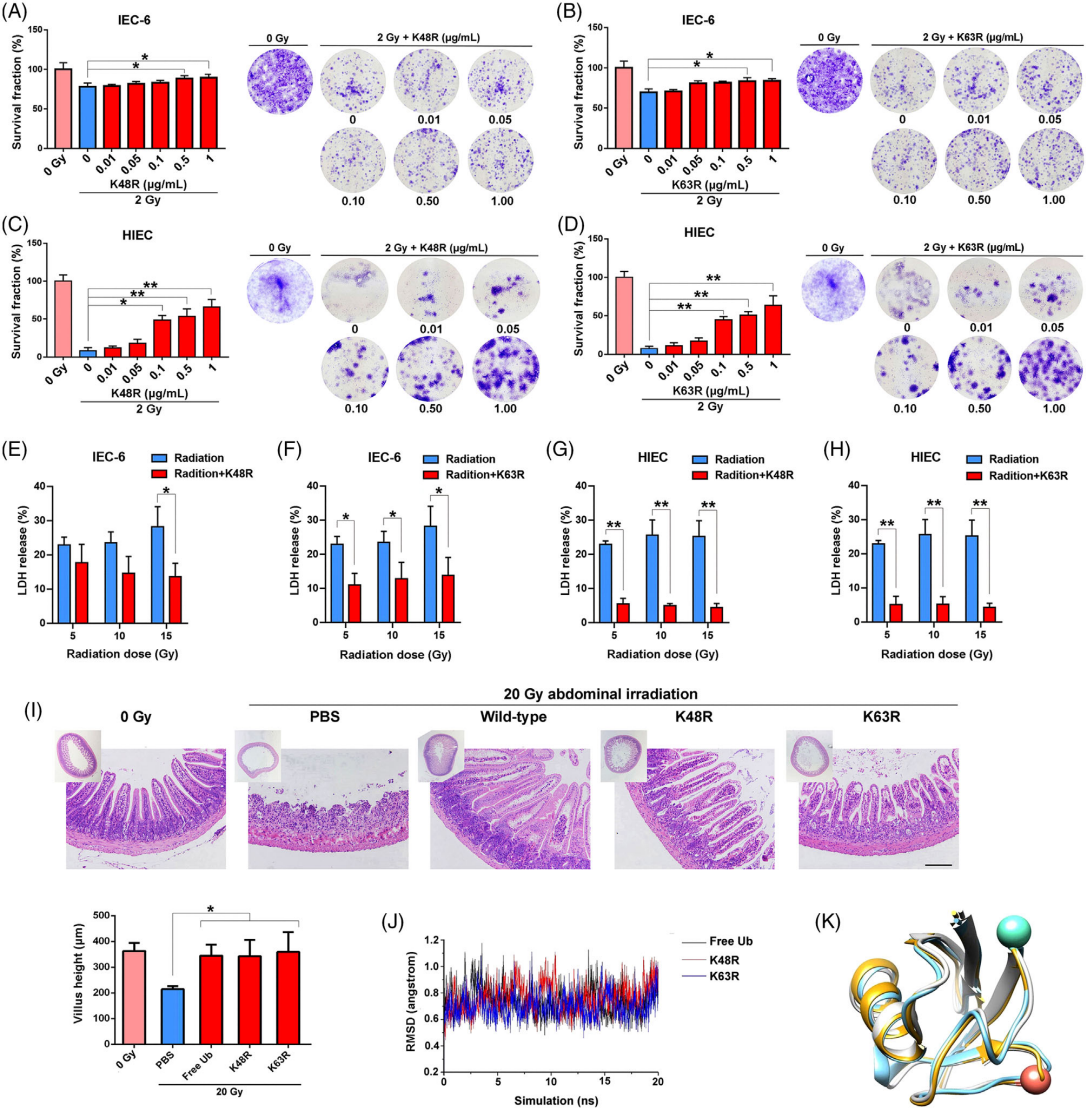
The effect of ubiquitin mutants on radiation‐induced intestinal injury. (A)–(D) Clonogenic cell survival assay was performed with IEC‐6 or human intestinal epithelial cells (HIEC) that were treated with indicated concentration of free ubiquitin mutants (K48R and K63R) for 24 h and then exposed to 2 Gy radiation. The survival fraction was normalized to the unirradiated control group. Representative clones from each group after 0 or 2 Gy radiation are shown. (E)–(H) After incubation of free ubiquitin mutants for 24 h, cells were irradiated and LDH of each group was measured. (I) Representative H&E‐stained sections of the small intestines. Mice were i.p. injected with PBS, wild‐type free ubiquitin or mutated free ubiquitin. Mice were then exposed to 20 Gy abdominal irradiation and small intestines were harvested for pathological examination 3.5 days post radiation (*n* = 4). Scale bar = 100 μm. (J) RMSD evolutions of wild‐type ubiquitin and K48R and K63R mutants throughout 20‐ns long simulations. (K) Superimposition of final structures of wild‐type (in blue), K48R (in white), and K63R (in gold) ubiquitin. Proteins are shown in ribbon. Position of K(R)48 (in pink) and K(R)63 (in light green) are also indicated by spheres

Since K48‐ and K63‐linked Ub chains are the two most abundant ubiquitination chain types,^26^ we next investigated whether Ub mutants lacking K48 or K63 affect the radioprotective role of free Ub. The effect of free Ub mutants (K48R and K63R) on the radiosensitivity of IEC‐6 cells was determined via clonogenic survival assay. As shown in Figure S4, Figure 6A,B and Figure S10, pretreatment with free Ub mutants significantly increased the survival rate and cell viability in a dose‐dependent manner in IEC‐6 and HIEC cells. Moreover, radiation‐induced cell death was alleviated by free Ub mutants (K48 or K63) pretreatment in IEC‐6 and HIEC cells following 5, 10, and 15 Gy X‐ray exposure (Figure 6E–H). In vivo study showed that Ub mutants (K48R and K63R) were as potent as free Ub in facilitating the recovery of small intestine after 20 Gy abdominal irradiation (Figure 6I). These results demonstrated that both K48 and K63 mutants of free Ub remained radioprotective and the covalent linkage of Ub was unlikely to be required during this process.

Molecular dynamics (MD) simulation was utilized to compare the structural alteration of wild‐type Ub and two Ub mutants. The three simulation systems were wild‐type Ub and K48R and K63R mutants. Each system was used to conduct a 20 ns simulation. Evolution of root mean square deviation (RMSD) throughout the simulation was calculated based on backbone atoms shown in Figure 6J. It is shown that, in all the systems, RMSD level off within a notably short period of time, indicating that they reach stable states quickly. The average RMSDs of wild‐type Ub, K48R protein, and K63R protein during the last 10 ns were 0.73, 0.71, and 0.71 Å, respectively. Such small RMSDs demonstrate the limited structural changes of all three proteins. Structural comparison was also performed by superimposing three final models as shown in Figure 6K. It is clear that the overall structures of three models are superimposed effectively without significant changes. Since non‐bond interactions, such as hydrogen bonds and salt bridges, are closely related to protein conformational change, ligand identification, and fundamental physiology functions, we investigated the salt bridges and hydrogen bonds formed by K(R)48 and K(R)63. In wild‐type Ub, K48 mainly formed hydrogen bonds to F45, and K63 formed salt bridge to E64. Both non‐bond interactions were well‐reserved in the two mutant systems. Additionally, K(R)48 occasionally formed salt bridges to E51 in the two mutant systems with very low survival percentage (< 4% during the entire simulation in both systems). Thus, both overall structure comparison and residue interaction analysis indicated that limited change occurs when K48 and K63 were mutated to Arginine.

### 2.8 Free ubiquitin promotes radioprotective fibroblast growth factor‐2 secretion in intestinal cells

**FIGURE 7:**
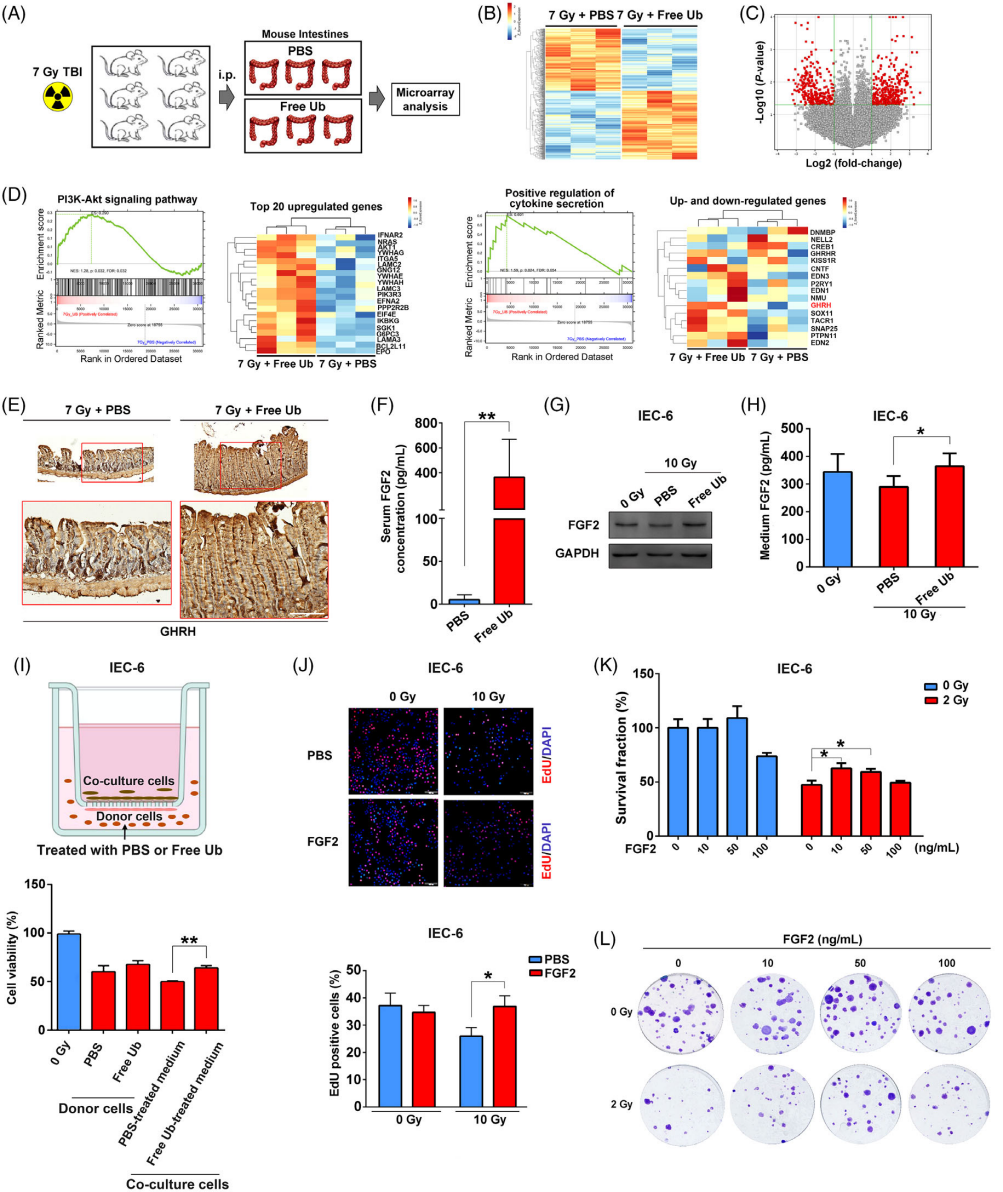
Free ubiquitin promotes FGF2 expression and secretion. (A) Experimental design of the microarray analysis. (B) Heatmap of gene clusters for those differentially expressed between PBS and free ubiquitin (Ub)‐treated mice at 12 h following 7 Gy total body irradiation (TBI). (C) Volcano plot comparing PBS and free Ub treatment. Genes with fold change > 2 and *P* value < 0.05 are marked with red dots and those with fold change < − 2 and *P* value < 0.05 are marked with green dots. (D) Significantly enriched pathways altered in mice 12 h post 7 Gy TBI (PBS vs. free Ub treatment). (E) IHC staining of GHRH in mouse intestine. Scale bar = 100 μm. (F) Mice were i.p. treated with PBS or free Ub before 7 Gy radiation. Serum concentration of FGF2 in mice was measured 5 days after radiation (*n* = 5). (G) Western blotting analysis of FGF2 expression in IEC‐6 cells. (H) Quantification of FGF2 levels in the culture medium with or without free Ub by ELISA. (I) IEC‐6 cells were pretreated with free Ub for 24 h. And then, the culture medium was changed followed by 10 Gy radiation. Then, cells were co‐cultured with IEC‐6 cells by a transwell chamber. Cell viability of irradiated (donor) cells and co‐cultured (receptor) cells were measured. (J) The effect of FGF2 (50 ng/ml) on the proliferation of IEC‐6 cells with or without radiation. Scale bar = 50 μm. (K) Clonogenic survival assay of IEC‐6 cells that were treated with indicated concentration of FGF2 for 24 h and then exposed to 0 or 2 Gy radiation. The survival fraction was normalized to the unirradiated control group. (L) Representative clones from each group after 0 or 2 Gy radiation. *P < 0.05 and **P < 0.01, compared with the control group

To unravel the underlying mechanisms of free Ub‐induced radioprotection of normal tissues, we conducted high‐throughput mRNA microarray analysis of mouse intestines following 7 Gy radiation with or without free Ub (Figure 7A). A serial of differentially expressed mRNAs between the two treatment groups were screened out (Figure 7B,C). The raw microarray data are accessible through Gene Expression Omnibus series accession number GSE133377. The expression of seven genes including *FKBPL*, *PDE7A*, and *VGLL1* was further validated by RT‐PCR, which was consistent with the microarray data (Figure S11). Using GSEA^27^ to screen differentially expressed pathways, PI3K‐Akt pathways were enriched (*P* = 0.032; Figure 7D), which was consistent with our previous results. Notably, this in silico analysis also revealed the enrichment of the cytokine secretion pathway (*P* = 0.024; Figure 7D), indicating that free Ub probably affected the key cytokine(s) to exert radioprotection. Growth hormone releasing hormone (GHRH), which has been shown to increase the expression of multiple cytokines such as FGF2 in cancerous and noncancerous cells,^28^, ^29^ was confirmed to be induced in free Ub‐treated mouse intestine (Figure 7D,E). We therefore examined the serum levels of 10 important cytokines that have been documented to be involved in tissue radiosensitivity, tissue regeneration, and/or cell proliferation.^1^, ^2^, ^3^, ^4^, ^5^, ^6^, ^7^, ^8^, ^9^, ^10^, ^11^, ^12^, ^13^, ^14^, ^15^, ^16^, ^17^, ^18^, ^19^, ^20^, ^21^, ^22^, ^23^, ^24^, ^25^, ^26^, ^27^, ^28^, ^29^, ^30^, ^31^, ^32^ As shown in Figure 7F, administration of free Ub following radiation significantly elevated serum levels of FGF2 but not the other nine cytokines (Figure S12).

Given that FGF2, a member of the FGF family, has been reported to be involved in a range of critical physiological processes—including embryonic development, cell proliferation, tissue repair, tumor growth, and metastasis,^33^, ^34^ we thus focused on how free Ub modulated FGF2 induction in response to radiation‐induced damage. In parallel with in vivo data, FGF2 expression (Figure 7G) and secretion were higher in free Ub‐treated IEC‐6 cells than that PBS‐treated cells following radiation (Figure 7H). Moreover, free Ub ‐treated medium maintained the cell viability of irradiated receptor IEC‐6 cells, suggesting a possible mechanism of free Ub through the secretion‐related pathway (Figure 7I). We further demonstrated that theFGF2 treatment facilitated the survival and proliferation of irradiated IEC‐6 cells (Figure 7J–L) with increased phosphorylation of Akt (Figure S13).

### 2.9 FGF2 signaling mediates the radioprotective role of free ubiquitin

**FIGURE 8:**
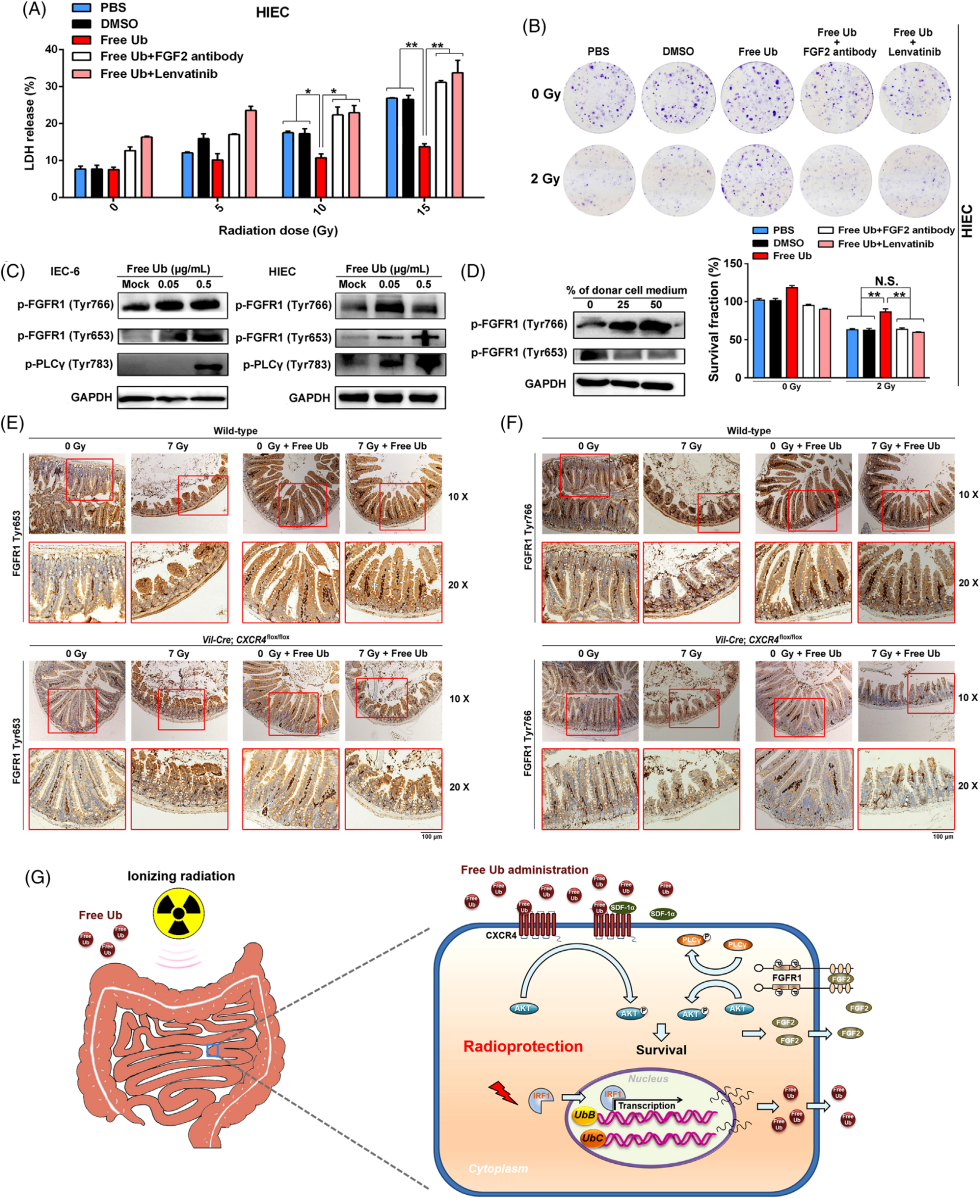
FGF2 signaling mediates the radioprotective role of free ubiquitin. (A) Blockade of FGF2 compromised the radioprotective role of free ubiquitin (Ub). Human intestinal epithelial cells (HIEC) were treated with an anti‐FGF antibody (R&D, Minneapolis, MN; #Ab‐233‐NA, 1 μg/mL) or lenvatinib (1 μM), together with free Ub. 24 h after ionizing radiation, LDH of each group was measured. *P < 0.05 and **P < 0.01 compared with the control group. (B) Clonogenic cell survival assay of HIEC cells treated with an anti‐FGF2 antibody (1 μg/ml) or lenvatinib (1 μM), together with free Ub, and then exposed to 0 or 2 Gy radiation. The data are presented as the mean ± SEM and normalized to the PBS‐treated cells. **P < 0.01, compared with the control cells. N.S., nonsignificant. (C) Western blotting analyses of FGFR1 phosphorylation (Tyr653 and Try 766) treated with free Ub after radiation. (D) IEC‐6 cells (donor cells) were pretreated with free Ub for 24 h. And then, the culture medium was changed followed by 10 Gy radiation. Western blotting analyses of FGFR1 phosphorylation (Tyr653 and Try766) in IEC‐6 cells treated with the culture medium of donor cells. (E) Representative images of IHC staining of FGFR1 Try 653 in intestinal tissues from *Vil‐Cre*; *CXCR4* ^flox/flox^ and control mice with or without radiation. Scale bar = 100 μm. (F) Representative images of IHC staining of FGFR1 Tyr766 in intestinal tissues from *Vil‐Cre*; *CXCR4* ^flox/flox^ and control mice with or without radiation. Scale bar = 100 μm. (G) Schematic representation of the radioprotective role of free ubiquitin. IRF1 is activated by ionizing radiation and promotes ubiquitin expression. Free ubiquitin (Ub) activates cellular membrane receptor CXCR4, which increases Akt phosphorylation and FGF2 secretion. FGF2 protects irradiated intestinal cells through its receptor FGFR1. Thus, free ubiquitin confers protection against radiation‐induced intestinal injury through the CXCR4/FGF2 axis

To confirm whether FGF2 mediated the radioprotective role of free Ub, the effects of anti‐FGF2 antibody and lenvatinib, a specific inhibitor of FGF receptors (FGFRs),^35^ on freeUb‐treated intestinal epithelial cells were examined by LDH release assay. As shown in Figure 8A, blockade of FGF2 by its antibody or its inhibitor abrogated the radioprotective role of free Ub as evidenced by increased LDH release following 10 or 15 Gy radiation. In line with this, clonogenic survival assay showed that inhibition of FGF2 signaling did not affect cell survival without radiation but counteracted the increased survival of free Ub following radiation in HIEC cells (Figure 8B). The above results indicate that FGF2 signaling mediated the radioprotection of free Ub.

Based on the above results, we sought to evaluate whether FGF2 downstream signaling was activated upon free Ub treatment. FGF‐2 has been shown to bind to FGF receptors FGFR1, FGFR2, and FGFR4.^36^ We focused on FGF2 tyrosine kinase receptor FGFR1, due to its ability to modulate cell radiotolerance through phospholipase C (PLCγ).^37^ Results from Western blotting analysis showed that free Ub increased the Tyr653 and Tyr766 phosphorylation of FGF2 tyrosine kinase receptor FGFR1, and phosphorylation Tyr783 of PLCγ in HIEC and IEC‐6 cells (Figure 8C). Moreover, the culture medium from free Ub ‐treated IEC‐6 cells resulted in phosphorylation of FGFR1 Tyr766 (Figure 8D), indicative of its activation on FGFR1 signaling. FGFR1 activation was then confirmed in wild‐type and *CXCR4* deficient mice treated with free Ub. The results from IHC staining revealed that both FGFR1 Tyr766 and Tyr653 phosphorylation were increased in mouse intestines upon free ubiquitin in vivo. Conditional deletion of CXCR4(*Vil‐Cre*; *CXCR4* ^flox/flox^) blunted FGFR1 Tyr653 and Tyr766 phosphorylation (Figure 8E,F), suggesting that FGFR1 activation depended on CXCR4. Taken together, these results indicated that free Ub activated FGF2/FGFR1 pathway through CRCR4, which enhanced the survival of irradiated intestinal epithelial cells.

## 3 DISCUSSION

Radiation‐induced acute injuries including intestinal injury remain a cause for significant concern. Although the search for suitable radiation countermeasures for radiation‐associated injuries was initiated over half a century ago, few safe and effective radiation countermeasures for these injuries have been approved by the Food and Drug Administration (FDA),^28^, ^38^ which limits clinical treatment options. The dearth of radiation countermeasures has prompted intensified research to achieve disease prevention or reversal.^6^ Therefore, it is necessary and valuable to identify novel molecules, especially naturally occurring molecules, that can be used to treat radiation‐induced injuries.

The ubiquitin/proteasome system (UPS) is conserved among all eukaryotes and plays vital regulatory roles in numerous cellular functions, including cell radiosensitivity.^20^ Although free Ub has been considered as a normal constituent in serum and bodily fluids,^8^, ^39^ its influence on tissue radiosensitivity has not yet been reported. To our knowledge, this is the first observation that free Ub confers dramatic protection against radiation‐induced death and intestinal injury, which is a previously unappreciated radioprotective strategy. Unlike the reported radioprotective drugs above, in vitro and in vivo administration of free Ub did not result in detectable adverse effects possibly because free Ub is an endogenous and innate peptide, indicating its promise in future clinical application. Moreover, radiation specifically upregulates the levels of free Ub in irradiated intestines, but not in other tissues such as lung, liver, and heart, indicating an intestine‐specific adaption and defensive response to ionizing radiation. In the present work, we elucidated that radiation‐induced free Ub was regulated by IRF1, which has been identified as a novel radiation‐responsive transcription factor and serves as a natural receptor of free Ub. IRF1 was likely to be activated by radiogenic cytoplasmic DNA/cGAS/STING pathway (our unpublished data). Radiation‐related transcriptional factors such as Nrf2, YY1, and β‐catenin are predicted to be involved in *UbB* or/and *UbC* transcription, however, they are yet to be verified.

CXCR4 on the plasma membrane has been identified as a natural receptor of free Ub in recent years.^19^, ^40^ Although SDF‐1α and free Ub are both reported ligands for CXCR4 and SDF‐1α and free Ub exert a synergistic effect in Akt activation,^18^ they interact with CXCR4 through distinct regions.^19^ These results suggest that free Ub and SDF‐1α might trigger distinct downstream effectors upon activating CXCR4 receptor, respectively.^19^ Consistent with this notion, the synergistic radioprotective effect was not observed in irradiated intestinal epithelial cells by applying SDF‐1α and free Ub. Free Ub alone sufficiently mitigated radiation‐induced tissue damage and promoted cell regeneration after radiation exposure via Akt signaling pathway. Akt is a well‐established target for radioprotection, which directly facilitates DNA repair and promotes cell survival.^41^ A variety of agents that can activate Akt has shown the radioprotective activity.^41^ Although CXCR4 was herein found to mediate the radioprotective function of free Ub, SDF‐1α is unlikely to exert similar radioprotective role as free Ub. SDF‐1α has also been shown to bind to another receptor CXCR7 with greater affinity than does CXCR4.^42^, ^43^ The significance of the SDF‐1α/CXCR7 axis in signaling transduction and the progression of various diseases have been widely characterized.^44^ Additionally, other possible receptors for free Ub cannot be excluded, which merits further investigation.

Through microarray‐based screening and bioinformatic analysis, the cytokine secretion‐related pathway was found to be enriched in free Ub treated mouse intestinal tissues post radiation. Increased serum FGF2 and activated FGFR1 were associated with free Ub treatment after radiation. FGF2, also known as basic FGF (bFGF), plays important roles in the regulation of cell division, angiogenesis, cell differentiation, migration, and cell survival under various physiological and pathological conditions by interacting with its receptors.^45^, ^46^ Specifically, FGF2 has been shown to cooperate with IL‐17 to accelerate proliferation and repair the damaged intestinal epithelium in a dextran sulfate sodium salt‐induced mouse colitis model.^47^ The FGF2 expression has been reported to be modulated by GHRH.^28^, ^29^ Nevertheless, the exact mechanism by which GHRH drives FGF2 has yet to be determined. FGF2 ligand binds the extracellular domain of FGFR1, which subsequently activates cytoplasmic tyrosine kinase domains by tyrosine autophosphorylation at multiple sites.^48^ FGF2 has been shown to mitigate radiation‐induced injuries alone or together with other treatment.^49^ FGF2‐induced downstream signaling mediated via FGFR1 has been shown to induce glioblastoma radioresistance through its downstream effector PLCγ,^36^ indicative a potential role of FGFR1 in modulating normal cell radiosensitivity. Herein, we show that free Ub promoted the phosphorylation of FGFR1 at Tyr653 and Tyr766, which is significant for its kinase activity and PLCγ interaction, respectively.^36^ We also showed that PLCγ1 phosphorylation was increased after free Ub treatment, which corresponded to FGFR1 Tyr766 phosphorylation. Of note, we are not ruling out the importance of other FGF2 receptors in contributing to FGF2‐mediated radioprotection. These results indicate that free Ub stimulated the downstream effectors of FGF2, which play a role in intestinal epithelium survival.

In conclusions, free Ub is a radiation‐responsive biomolecule that performs the radioprotective roles against radiation‐induced intestinal injury through the CXCR4/FGF2 axis (Figure 8G). However, further in‐depth investigations to elucidate the free Ub‐associated regulatory mechanisms in radiation‐induced intestinal injury are indispensable. First, the relationship between intracellular Ub levels and systemic Ub concentrations in context of radiation‐induced normal tissue injury is still unclear, which is worth further in‐depth mechanistic studies. Second, it remains unclear whether and how the exogenous free Ub may affect the expression and secretion of endogenous free Ub, which is an important issue for the development of free Ub as a novel radioprotective drug. And we are considering to carefully carry out the well‐designed pharmacological and pharmacodynamic studies in the following study. Third, the optimal dose and dosage of free Ub as a radioprotective drug have not been decided, which will be another research direction in the future. Nevertheless, the present study still provides a novel and promising therapeutic option for preventing the intestinal side effects of radiotherapy and treating victims of incidental radiation exposure.

## 4 MATERIALS AND METHODS

### 4.1 Reagents and materials

Free Ub, di‐Ub, and Ub mutants (K48 and K63) were purchased from R&D Systems (Minneapolis, MN) and Ubbiotech (Changchun, China). Recombinant FGF2 and SDF‐1α were obtained from PrimeGene Biotech (Shanghai, China). Lenvatinib (E7080) was obtained from MedChemExpress (MCE, Monmouth Junction, NJ). 4′‐6‐Diamidino‐2‐phenylindole (DAPI) and tamoxifen were purchased from Sigma–Aldrich (St. Louis, MO). Control adenovirus, an *IRF1* overexpression adenovirus (Ad‐IRF1), control lentivirus (Lenti‐NC), and lentiviruses targeting IRF1(Lenti‐IRF1) were obtained from HanBio (Shanghai, China).

### 4.2 Mice and irradiation

Protocols for experiments involving animals were approved by the Animal Experimentation Ethics Committee at Soochow University (Suzhou, China). Male C57BL/6J mice (8 weeks old, weighing 23–26 g) were purchased from Shanghai SLAC Laboratory Animal Co., Ltd. (Shanghai, China). The C57BL/6 *IRF1* knockout (*IRF* ^–/–^) mice were obtained from The Jackson Laboratory (stock number: 002762). Animals were housed under controlled conditions of 20°C–22°C and relative humidity of 50%, a fixed 12 h light/dark cycle, and ad libitum access to food and water. For TBI, mice were irradiated using a 6‐MV accelerator (Clinac 2100EX; Varian Medical Systems, Palo Alto, CA) at a fixed rate of 200 cGy/min. For abdominal irradiation, mice were anesthetized with an intraperitoneal (i.p.) injection of 3.5% chloral hydrate (0.1 ml/10 g body weight) and placed on a platform to receive 20 Gy abdominal irradiation at a fixed rate of 200 cGy/min. The irradiated field was from the xiphoid process to the pubic symphysis.

To obtain mice with *CXCR4* specifically knocked out in the intestine, we used Cre/loxP‐mediated recombination for conditional gene targeting. *CXCR4* flox mice were obtained from Jackson Laboratory (stock number 008767), which possess loxP sites on either side of exon 2 of the Cxcr4. To perform intestine‐specific knockout of *CXCR4*, we used *Vil1*‐cre mice expressing Cre recombinase *in villus* and crypt epithelial cells of the small and large intestines (stock number 021504). Cre expression was induced by tamoxifen. *Vil‐CXCR4* ^flox/flox^ and control mice (*CXCR4* ^flox/flox^) were treated daily with 0.1 ml of a 10 mg/ml tamoxifen ethanol corn oil solution for 5 consecutive days at 8 weeks of age. The genotypes of the mice were confirmed using PCR, following the Jackson Laboratory instructions.

To investigate the radioprotective role of free Ub, mice were randomly divided into two groups: (1) i.p. injection of 100‐μl volume PBS; (2) i.p. injection of free Ub in a 100‐μl volume PBS (3–10 mg free Ub /kg animal body weight). Mice were exposed to indicate doses of radiation 1 h after injection unless otherwise stated.

### 4.3 Intestinal organoid isolation and culture

The C57BL/6J mice were sacrificed, and the small intestines were dissected. Intestinal organoid was isolated according to the previous reported methods by Li et al.^50^ In brief, after gently washing with cold PBS, the intestines are cut into 1–2 mm long pieces and incubated with Gentle Cell Dissociation Reagent (Stem Cell Technologies, Vancouver, BC, Canada) at room temperature (15°C–25°C) for 15 min on a rocking. The suspension was filtered through a 70 μm cell strainer (BD Biosciences, San Diego, CA) to remove the residual villous material. The pelleted crypts were re‐suspended in complete IntestiCult™ Organoid Growth Medium (Stem Cell Technologies) at the density of 500 crypts per 50 μl. The cell suspension was then mixed with the organoid matrix (Organoid Tech., Chengdu, China) and seeded in pre‐warmed 12‐well plates. The organoids were incubated with Organoid Growth Medium containing 0, 0.5 and 1 μg/ml free Ub. 24 h later, the crypts were subjected to 10 Gy X‐ray radiation and cultured continuously at 37°C in 5% CO_2_. Images were taken on days 0, 6, and 12 of culture.

### 4.4 Cell cultures and irradiation

Rat jejunal crypt cell line IEC‐6 and HIEC line were purchased from the Cell Bank of the Chinese Academy of Sciences (Shanghai, China) as reported previously.^51^, ^52^ The cells were placed in Dulbecco's modified Eagle's medium (DMEM) supplemented with 10% FBS (v/v; Gibco, Grand Island, NY) and 1% (v/v) penicillin–streptomycin (Beyotine, Nantong, China) at 37°C with 5% CO_2_. For IEC‐6 cells, 10 μg/ml recombinant human EGF (Gibco) and 10 μg/ml bovine insulin (Yeasen, Shanghai, China) were added. For ionizing irradiation, an X‐ray linear accelerator (RadSource, Suwanee, GA) at a fixed dose rate of 1.15 Gy/min was applied.

### 4.5 Luciferase assay

The IRF1‐responsive luciferase reporter was obtained from Qiagen. Luciferase reporters with wild‐type or IRF1 binding sites deleted *UbB* and *UbC* promoters were constructed by PPL Biotech (Nanjing, China) and confirmed by sequencing. Cells were transfected with reporter vectors using Fugene HD transfection reagent (Promega, Madison, WI). In each transfection, pRL‐TK (Promega) was used to correct for the transfection efficiency. The Luciferase activity was measured with the Dual‐Luciferase Reporter Assay System (Promega). The promoter activity was expressed as the ratio of *firefly* luciferase to *Renilla* luciferase activity.

### 4.6 Clonogenic survival assay

Clonogenic survival assay of intestinal cells was performed as reported previously.^52^ IEC‐6 and HIEC cells were pretreated with free Ub or its mutants for 24 h and then exposed to 0 or 2 Gy X‐ray radiation. After the irradiation, the cells were grown from 7–10 days to allow for colony formation and were subsequently fixed and stained using crystal violet. Colonies consisting of 50 or more cells were counted as a clone. Relative clonogenic survival fraction was calculated.

### 4.7 Cell viability assay

Cells were seeded in a 96‐well plate at a density of 1×10^4^ cells per well. Cells were then treated with indicated concentration of free Ub and/or radiation. In vitro viability was measured using the Cell Counting Kit‐8(CCK‐8) (Dojindo Laboratories, Kumamoto, Japan). Optical density was measured at 450 nm using a micro‐plate reader (Biotek, Winooski, VT).

### 4.8 Lactate dehydrogenase release assay

Cell death was measured by the amount of lactate dehydrogenase (LDH) released into the supernatant using an LDH cytotoxicity assay kit (Beyotime, Nantong, China) according to the manufacturer's instructions. The results were presented as mean ± SEM of absorbance measured at 490 nm using a microplate reader (Biotek, Winooski, VT).

### 4.9 Statistical analysis

Data from multiple experiments were displayed as mean ± SEM. Cell viability, LDH release, and survival fraction experiment data were displayed as mean ± SD. Multiple treatments were analyzed by one‐way ANOVA followed by Tukey's test multiple comparisons test. Student's *t*‐test was used for comparing two groups. Survival curves were assessed based on the Kaplan–Meier method and compared using the log‐rank test. Gene expression results were compared by paired Student's *t*‐test. Statistical analyses were performed using PRISM version 6.0 (Graph‐Pad Software, San Diego, CA). The differences were considered significant at *P* < 0.05.

Other methods are detailed in the Supporting information.

## CONFLICT OF INTEREST

The authors declare that they have no competing interests.

## AUTHOR CONTRIBUTIONS

Shuyu Zhang and Jianping Cao conceived and designed the study. Yang Jiao, Jing Xu, Ailing Wu, Bin Song, Fenghao Geng, Ying Xu, and Congzhao Zhao carried out the molecular biology studies. Wei Zhu, Shuyu Zhang, and Jianping Cao drafted the manuscript and the figures. Jing Xu, Lu Pan, Min Hong, and Xiaoqian Li performed the animal experiments. Judong Luo, Pengfei Liu , and Ming Li collected serum samples. Wei Zhu and Yang Jiao performed the statistical analysis. Xuanyu Meng performed molecular dynamics simulations. Yang Jiao, Jianping Cao, and Shuyu Zhang modified the manuscript. All authors read and approved the final manuscript.

## ETHICS STATEMENT

The study was conducted under a protocol approved by the Ethics Committee of Soochow University. All patients provided signed, informed consent for their blood to be used for scientific research.

## Supporting information

Supporting Information

## Data Availability

The raw microarray data are accessible through Gene Expression Omnibus series accession number GSE133377. Other data are available with reasonable request.
