# Supplementary material for: Interferon regulatory factor 1‐triggered free ubiquitin protects the intestines against radiation‐induced injury via CXCR4/FGF2 signaling
Source: MedComm (2020). 2022 Aug 26;3(3):e168. doi: 10.1002/mco2.168 (PMC9416916; doi:10.1002/mco2.168)
Supplement: Supplementary file 1 — Supporting Information [file MCO2-3-e168-s001.pdf]

IRF1-triggered free ubiquitin protects the intestines against radiation-induced injury via CXCR4/FGF2 signaling

Yang Jiao[a,b], Jing Xu[a,b], Bin Song[c], Ailing Wu[d], Lu Pan[a,b], Ying Xu[a,b], Fenghao Geng[d], Xiaoqian Li[e], Congzhao Zhao[a,b], Min Hong[a,b], Xuanyu Meng[a,b], Judong Luo[f], Pengfei Liu[a], Ming Li[a,b], Wei Zhu[a,b], Jianping Cao[a,b,*], Shuyu Zhang[c,d,e,f,g,*]

[a]School of Radiation Medicine and Protection, Medical College of Soochow University, Suzhou 215123, China.

[b]State Key Laboratory of Radiation Medicine and Protection and Collaborative Innovation Center of Radiation Medicine of Jiangsu Higher Education Institutions, Soochow University, Suzhou 215123, China.

[c]Laboratory of radiation Medicine, West China Second University Hospital, Sichuan University, Chengdu 610041, China.

[d]Second Affiliated Hospital of Chengdu Medical College, China National Nuclear Corporation 416 Hospital, Chengdu 610051, China.

[e]West China School of Basic Medical Sciences & Forensic Medicine, Sichuan University, Chengdu 610041, China.

[f]Department of Oncology, The Affiliated Changzhou No. 2 People's Hospital of Nanjing Medical University, Changzhou 213004, China.

[g]NHC Key Laboratory of Nuclear Technology Medical Transformation (Mianyang Central Hospital), Mianyang 621099, China.

***Corresponding authors**:

Zhang S, No. 17 South People's Rd, West China Second University Hospital, Sichuan University, Chengdu 610041, China. Tel./Fax:+86-28-85502429; E-mail: zhang.shuyu@hotmail.com or zhangshuyu@scu.edu.cn.

Cao J, No. 199 Ren'ai Rd, Medical College of Soochow University, Suzhou 215123, China. Tel./Fax:+86-512-65880037; E-mail: jpcao@suda.edu.cn.

Supplementary Materials and Methods

Monkeys and serum samples

The rhesus monkeys (*Macaca mulatta*) were housed at Beijing Institute of Radiation Medicine (Beijing, China). The serum of rhesus monkeys (n = 6) was received as gifts from Prof. Ming Li. The monkeys were exposed to 7 Gy γ-radiation at a dose rate 1.3 Gy/min using a ^{60}Co source. Monkey serum was collected before and after radiation exposure. Housing, treatment and veterinary care for the monkeys used in the study were approved by the Animal Experimentation Ethics Committee at Beijing Institute of Radiation Medicine. The monkeys weighed between 4.0 and 6.5 kg and were aged ~5 years old. The monkeys were anesthetized by intravenous injection of 3% sodium pentobarbital (30 mg/kg). Body temperature was maintained at 37 °C. After radiation exposure, there was no pain and uncomfort for the animals.

Patients and serum samples

Serum samples of cancer patients were obtained from Changzhou Cancer Hospital affiliated to Soochow University (Changzhou, China) between June 2012 and March 2014 as reported previously. The samples were obtained from 11 esophageal cancer patients, 4 lung cancer patients and 5 rectal cancer patients. Venous blood was drawn from the 20 volunteers before radiotherapy, during radiotherapy (after 15 fractions of radiotherapy; 30 Gy), and 24 h after radiotherapy (60 Gy). All collected blood samples were stored at room temperature for 60 min. After centrifugation at 3000 × g for 10 min, supernatants were collected and immediately frozen at −80 °C to preserve them until further use. All patients provided signed, informed consent for their blood to be used for scientific research. Ethical approval for this study was obtained from Changzhou Cancer Hospital Affiliated to

Soochow University. All experiments were performed in accordance with the relevant guidelines and regulations of Soochow University.

Immunofluorescence assay

HIEC and IEC-6 cells were fixed with 4% paraformaldehyde, washed with PBS, and permeabilized with 1% Triton X-100 in PBS. Cells were blocked with blocking buffer (PBS, 1% Triton X-100, and 5% BSA) and incubated at 4°C with antibodies against IRF1 (Cell Signaling Technology, Beverly, MA; #8478S) overnight. FITC-conjugated goat anti-mouse (1:100) was added for 30 min at room temperature. Nuclei were counterstained with DAPI.

Blood cell count

20 μL ocular blood was drawn from mice using a centrifuge tube with EDTA-2Na. Blood cell counts (leucocytes/WBC, erythrocytes-RBC, hemoglobin-HGB, and thrombocytes-PLT) were determined using a hemocounter (XE2100; Sysmex Co., Kobe, Japan).

Histology analysis and immunohistochemistry (IHC)

Tissues were isolated at different time points after irradiation, fixed in 10% neutral formalin overnight, and embedded in paraffin; then, 3 μm-thick paraffin sections were stained with hematoxylin and eosin (H&E) or other staining reagents. Images of tissue sections were captured using an Olympus optical microscope (Olympus Corp., Tokyo, Japan).

For IHC analysis, 3 μm-thick paraffin sections were deparaffinized, rehydrated, and incubated with 3% hydrogen peroxide for 15 min at room temperature to block endogenous peroxidase. Heat-mediated antigen retrieval was performed in sodium citrate buffer (pH 6.0) for 3 min using a pressure cooker. For blocking non-specific-binding sites, the sections were incubated with 5% bovine serum albumin (BSA) for 30 min at room temperature. Then, the primary antibodies against Ki67 (Beyotime, Nantong, China), Akt (Abcam,

Cambridge, MA; #Ab8805), p-Akt (Cell Signaling Technology, Beverly, MA; #3787), CXCR4 (Abcam, Cambridge, MA; #Ab124824), GNRH (GeneTex, Irvine, CA, #GTX64650), p-FGFR1 (Tyr 653, Abcam, Cambridge, MA; #Ab173305) and p-FGFR1 (Tyr 766, Abcam, Cambridge, MA; #Ab59180) were each diluted following the manufacturer's instructions and added to the sections and incubated overnight at 4 °C. Next, secondary antibody dilution (Zhongshan Golden Bridge Biotechnology, Beijing, China) was added and incubated at 37 °C for 1 h. Finally, the chromogenic reaction was developed with DAB and counterstained with hematoxylin.

Enzyme-linked immunosorbent assay (ELISA)

Free ubiquitin and FGF2 in serum or cell culture medium were measured using a commercial ELISA kit (Abnova, Taiwan, China). The protein concentration was calculated using a standard curve obtained from measuring the absorbance of known concentrations of highly purified recombinant proteins and expressed in pg/mL. Briefly, 0.5 mL of conditioned medium was centrifuged at 12000 ×*g* for 10 min at 4 °C, and the supernatant was stored at -80 °C for 72 h. Thawed supernatant (100 μL) was used in the ELISA kit according to the manufacturer's instructions. The optical absorbance of each sample was measured at 450 nm.

Quantification of cytokines in serum and tissues

Serum and tissue Interleukin-1β (IL-1β), IL-6, epidermal growth factor (EGF), endothelin-1, hepatocyte growth factor (HGF), G-CSF, fibroblast growth factors-2 (FGF2), VEGF-C, monocyte chemotactic protein 1 (MCP-1), and SDF-1α levels were measured by a Luminex 200 protein liquid chip platform (Millipore, Billerica, MA) according to the manufacturer's instructions. A standard serial dilution was included in each assay to obtain a standard

curve from which the sample concentration of the cytokines could be calculated using the measured absorbance value.

Reverse transcriptase-PCR (RT-PCR) and real-time PCR

Total RNA from mouse tissues was extracted with Trizol (Invitrogen, Carlsbad, CA) and reverse transcribed to cDNA using an oligo(dT)$_{12}$ primer and Superscript II (Invitrogen). The mRNA levels of target genes and internal standard *GAPDH* or *β-Actin* were measured by RT-PCR and real-time PCR in triplicate on a Prism 7500 real-time PCR machine (Applied Biosystems, Foster City, CA). The primers were listed in Supplementary Table 1.

Supplementary Table 1. Primers for RT-PCR analysis

Gene	Forward primer	Reverse primer
**Human *UbB***	5'-GGTGAGCTTGTTTGTGT CCCTGT-3'	5'-TCCACCTCAAGGGTGATGGTC-3'
**Human *UbC***	5'-TGCACCTGGTACTCCGTC TCA-3'	5'-CAGTGAGTGTCTTCACGAAGATTTG-3'
**Human *GAPDH***	5'-GCACCGTCAAGGCTGAGAAC-3'	5'-GGATCTCGCTCCTGGAAGATG-3'
**Rat *UbB***	5-TCTTCAGTCTGCATTCCCGG-3'	5'-CTAGAGTGCAGAGTAATGCCATCAG-3'
**Rat *UbC***	5'-ATCTAGAAAGAGCCCTTCTTGTGC-3'	5'-ACACCTCCCCATCAAACCC-3'
**Rat *GAPDH***	5'-GGCACAGTCAAGGCTGAGAATG-3'	5-ATGGTGGTGAAGACGCCAGTA -3'
**Mouse *UbB***	5'-CGGTCTTTCTGTGAGGGTGT-3'	5'-TTCACGTTCTCGATGGTGTC-3'
**Mouse *UbC***	5'- GGCATGCAGATCTTTGTGAA-3'	5'- TTCAAAGTGCAATGAAACTTGT-3'

Mouse *FKBPL*	5'-CCTTCCAGGCCTCAACATCA-3'	5'-GGCTGATAGGTGAGGTCTCC-3'
Mouse *IFT81*	5'-GAACTTCTGAAGCAGCGTCA-3'	5'-ACATGTCATCCAGGGTCCG-3'
Mouse *PDE7A*	5'-TGGCCTTGAAATGTGCTGAT-3'	5'-TGACACATGGAAAGCTTGCT-3'
Mouse *SLC181*	5'-TGGGAGTGTCTATGCCATCG-3'	5'-CTCCTCCTCCTTAGCTGGTG-3'
Mouse *VGLL1*	5'-TCCTGCTCAGTTTCCTACCA-3'	5'-ACCATTTTCAGGAGATGCTGC-3'
Mouse *Dusp2*	5'-GGAGATAAGTGCCTGGTTCC-3'	5'-GACTCCCCGACGTTGCTTAA-3'
Mouse *β-Actin*	5'-TGCTGTCCCTGTATGCCT-3'	5'-TTGATGTCACGCACGATTTC-3'

BrdU incorporation assay

The cell proliferation rate was determined by the uptake of 5-bromo-2′deoxyuridine-5′monophosphate (BrdU) into DNA. Cells in the logarithmic growth phase were trypsinized and transferred to chamber slides. Cells were treated with $ZnCl_2$ for 30 min. After 24 h, the cells were labeled with 10 μM BrdU (Sigma-Aldrich, St. Louis, MO) for 1 h. Then, the cells were fixed and permeabilized with 0.5% Triton X-100 and 0.1% citric acid. The cells were blocked in 5% bovine serum albumin (BSA), and immunofluorescence was performed as previously described [1] by incubating samples with anti-BrdU antibody (Abcam, Cambridge, MA) for 12 h at 4 °C. The cells were then washed with cold PBS and incubated with TRITC conjugated goat anti-rabbit IgG (Beyotime, Nantong, China) for 45 min at 37 °C. The cells were counter-stained with DAPI to demarcate nuclei and observed under a

fluorescence microscope (Olympus, Tokyo, Japan).

Microarray analysis and bioinformatic analysis

Microarray-based mRNA expression profiling was performed using the Illumina MouseWG-6_v2 software according to standard protocols (Illumina, San Diego, CA). Raw data were processed and cubic-spline normalized using the GenomeStudio software (Illumina). The microarrays contained approximately 45,200 assay probes corresponding to the annotated mouse mRNA sequences. Total RNA labeling and hybridization were performed in standard conditions according to manufacturer instructions. To identify differentially expressed genes, Fold Change (FC) filtering was performed with FC > 2.0 and P value < 0.05 as the cut-off. Hierarchical Clustering was performed to show distinguishable gene expression profiling among samples. Gene Set Enrichment Analysis (GSEA) [2] was applied to explore the specific gene module and regulatory network related to free ubiquitin-induced radioprotection in mouse intestine. The microarray analysis and bioinformatic analysis were carried out at Genenergy Biotech (Shanghai, China). The raw microarray data are accessible through Gene Expression Omnibus series accession number GSE133377.

Western blotting assay

Cells were lysed in RIPA buffer (150 mM NaCl, 100 mM Tris, pH 8.0, 1% Triton X-100, 5 mM EDTA, and 10 mM NaF) supplemented with 1 mM sodium vanadate, 2 μM leupeptin, 1 mM phenylmethylsulfonyl fluoride, 1 mM dithiothreitol, and 2 μM pepstatin A. After centrifugation at 4 °C for 10 min (12000 × g), the supernatant was collected and analyzed with western blotting. Nuclear proteins were extracted using the nuclear protein isolation kit (Beyotime, Nantong, China); 30 μg protein was fractionated by 10% SDS-PAGE and

electrophoretically transferred to polyvinylidene difluoride membranes (Millipore, Bedford, MA). After blocking with 5% nonfat milk in PBS-Tween-20 for 1 h at room temperature, the membranes were blotted with antibodies against ubiquitin (Santa Cruz Biotechnology, Santa Cruz, CA; #sc-16655), Akt (Abcam, Cambridge, MA; #Ab8805), p-Akt (Cell Signaling Technology, Beverly, MA; #3787), p-FGFR1 (Tyr 653, Abcam, Cambridge, MA; #Ab173305), p-FGFR1 (Tyr 766, Abcam, Cambridge, MA; #Ab59180), PLCγ1 (Cell Signaling Technology, Beverly, MA; #2822) and p-PLCγ1 (Tyr 783, Cell Signaling Technology, Beverly, MA; #2821). Cell apoptosis/necrosis antibody sample kit with apoptosis and necrosis related antibodies were obtained from Cell Signaling Technology (Beverly, MA; #9257T). Cell pyroptosis antibody sample kit with pyroptosis related antibodies were obtained from Cell Signaling Technology (Beverly, MA; #43811T). GAPDH (Beyotime, Nantong, China) and Lamin B1 (Proteintech, Rosemont, IL, 66095-1-lg) were used as a loading control. After washing 4 times with TBST, the membranes were incubated with a horseradish peroxidase (HRP)-conjugated anti-rabbit or anti-mouse secondary antibody (Santa Cruz Technology, Santa Cruz, CA) for 2 h. Protein bands were visualized and photographed using a FluroChem M imaging system (Proteinsimple, San Jose, CA).

Nude mice xenografts

Male BALB/C nude mice (6-8 weeks of age) were purchased from the Shanghai SLAC Laboratory Animal Co., Ltd. (Shanghai, China). Mice were maintained in a pressurized ventilated cage according to institutional regulations. The hind limbs of nude mice were grafted with HCT8 cells (1×10^6) in 100 μL of PBS via a subcutaneous injection. Mice were randomly divided into 4 groups (n = 8) and received one of the following treatments: (1)

intraperitoneal injection of normal saline on day 15; (2) intraperitoneal injection of free Ub (3 mg/kg in 100 μL PBS) on day 15; (3) radiation group (IR) with 10 Gy X-ray irradiation on day 15; (4) radiation plus free Ub group: intraperitoneal injection of free Ub (3 mg/kg in 100 μL PBS) followed by 10 Gy X-ray irradiation on day 15. The body weight and tumor volume of nude mice were measured at 2-day intervals for four weeks. On day 30, the animals were sacrificed and tumors were resected. Protocols for experiments involving animals were approved by the Animal Experimentation Ethics Committee at Soochow University.

Molecular dynamics simulations

Single point mutants K48R and K63R were introduced on the wildtype (wt) crystal structure of ubiquitin (PDB:2PE9) by using Modeler v9.5 [3], respectively. Three simulation systems were setup: wt, K48R and K63R. For each system, protein was solvated in 0.15 M NaCl. The TIP3 water model was used [4]. GROMACS v4.6.5 [5] was used to conduct the simulation with the CHARMM36 force field [6,7]. Long range electrostatics were calculated using the particle mesh Ewald (PME) method [8] with a 12 Å cut-off. Van der Waal interactions were modeled using Lennard-Jones 6–12 potentials with a 14 Å cut-off. All production runs were performed with the NPT ensemble, with a constant temperature at 300 K using the v-rescale thermostats [9], and a constant isotropic pressure at 1 bar using the Berendsen barostats [10]. All bonds were constrained with the LINCS algorithm [11]. The time step was set to 2 fs and the neighboring list was updated at every 10 time steps. Prior to production runs, energy minimization of 3000 steps with steepest descent method were carried out on each system followed by a 0.1 ns equilibration process in which position restrain was applied to the backbone of the protein. Production runs with lengths of 20 ns were conducted and coordinates were saved every 10 ps for analysis. VMD [12]

and Chimera [13] programs were used for visualization.

References

[1] Gu Q, He Y, Ji J, Yao Y, Shen W, Luo J, et al. Hypoxia-inducible factor 1alpha (HIF-1alpha) and reactive oxygen species (ROS) mediates radiation-induced invasiveness through the SDF-1alpha/CXCR4 pathway in non-small cell lung carcinoma cells. Oncotarget 2015;6:10893-907.

[2] Keller A, Backes C, Gerasch A, Kaufmann M, Kohlbacher O, Meese E, et al. A novel algorithm for detecting differentially regulated paths based on gene set enrichment analysis. Bioinformatics 2009;25:2787-94.

[3] Sali A, Potterton L, Yuan F, van Vlijmen H, Karplus M. Evaluation of comparative protein modeling by MODELLER. Proteins. 1995;23(3):318-26.

[4] Jorgensen WL, Chandrasekhar J, Madura JD, Impey RW, Klein ML. Comparison of simple potential functions for simulating liquid water. J Chem Phys. 1983;79:926-35.

[5] Hess B, Kutzner C, van der Spoel D, Lindahl E. GROMACS 4: Algorithms for highly efficient, load-balanced, and scalable molecular simulation. Journal of Chemical Theory and Computation. 2008;4(3):435-47.

[6] Klauda JB, Venable RM, Freites JA, O'Connor JW, Tobias DJ, Mondragon-Ramirez C, Vorobyov I, MacKerell Jr AD, Pastor RW. Update of the CHARMM all-atom additive force field for lipids: validation on six lipid types. The journal of physical chemistry B. 2010;114(23):7830-43.

[7] Best RB, Zhu X, Shim J, Lopes PE, Mittal J, Feig M, MacKerell Jr AD. Optimization of the additive CHARMM all-atom protein force field targeting improved sampling of the

backbone ϕ, ψ and side-chain χ1 and χ2 dihedral angles. Journal of chemical theory and computation. 2012;8(9):3257-73.

[8] Darden TA, York DM, Pedersen LG. Particle mesh Ewald: An NlogN method for Ewald sums in large systems. J Chem Phys. 1993;98:10089-92.

[9] Bussi G, Donadio D, Parrinello M. Canonical sampling through velocity rescaling. The Journal of Chemical Physics. 2007;126(1):014101.

[10] Berendsen HJC, Postma JPM, Vangunsteren WF, Dinola A, Haak JR. Molecular-Dynamics with Coupling to an External Bath. Journal of Chemical Physics. 1984;81(8):3684-90.

[11] Hess B, Bekker H, Berendsen HJC, Fraaije JGEM. LINCS: A linear constraint solver for molecular simulations. Journal of Computational Chemistry. 1997;18(12):1463-72.

[12] Humphrey W, Dalke A, Schulten K. VMD: Visual molecular dynamics. Journal of Molecular Graphics. 1996;14(1):33-&.

[13] Pettersen EF, Goddard TD, Huang CC, Couch GS, Greenblatt DM, Meng EC, Ferrin TE. UCSF Chimera—A visualization system for exploratory research and analysis. Journal of Computational Chemistry. 2004;25(13):1605-12.

Supplementary figures and figure legends

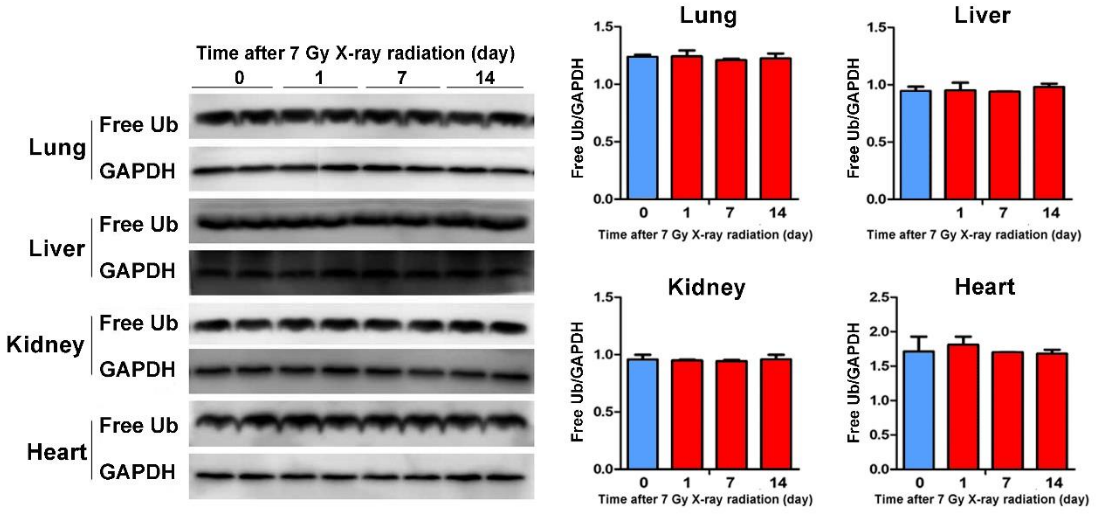

Figure S1. Western blotting analyses of ubiquitin expression in lung, heart, kidney, and liver tissues after radiation. Relative ubiquitin protein levels were calculated by Image J software. Data are presented as mean ± SEM.

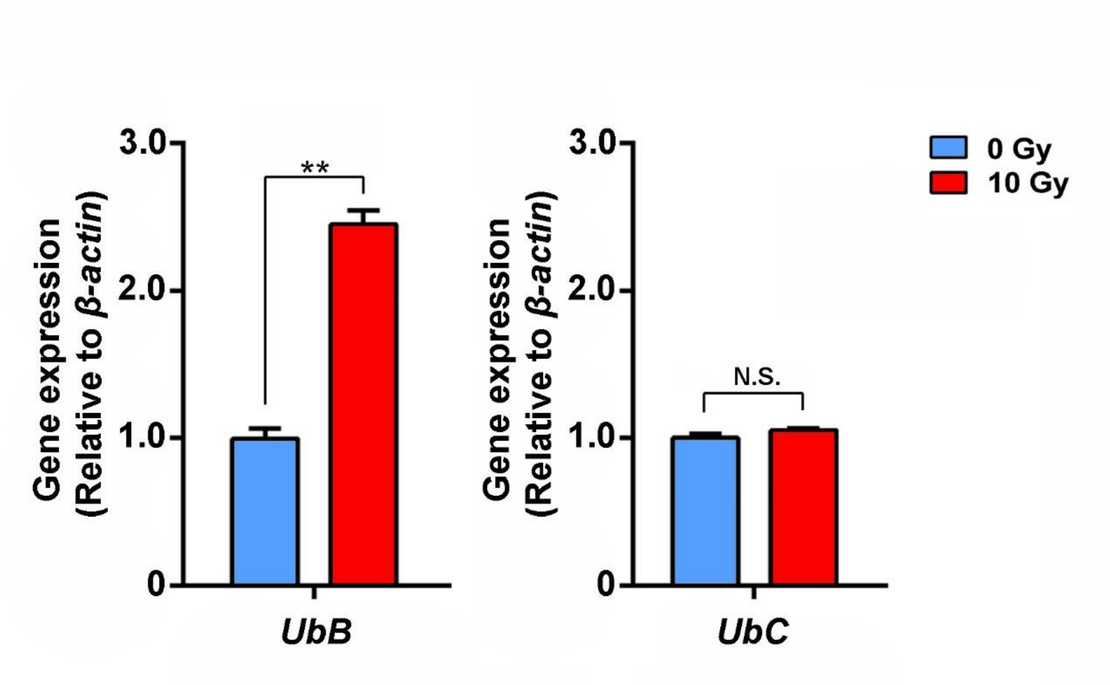

Figure S2. Real-time PCR analyses of the mRNA levels of ubiquitin coding genes *UbB* and *UbC* with or without radiation in mouse intestine tissues. Tissues were collected 24 h after radiation. Data are presented as mean ± SEM. ** *P* < 0.01 compared with the control group; N.S., non-significant.

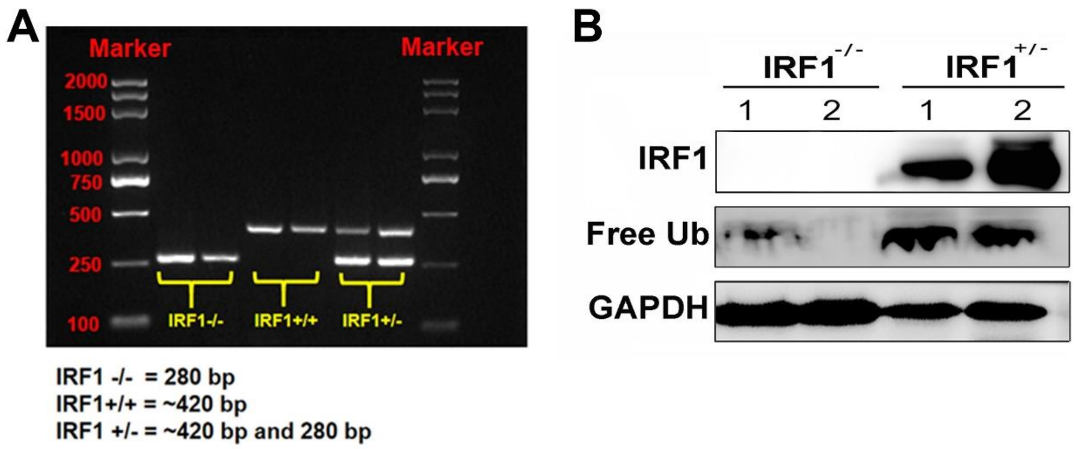

Figure S3. (A) PCR analysis of the genotypes of *IRF1* wild-type, heterozygous and deficient mice. (B) Western blotting analysis of free Ub expression in intestines of *IRF1* heterozygous and deficient mice.

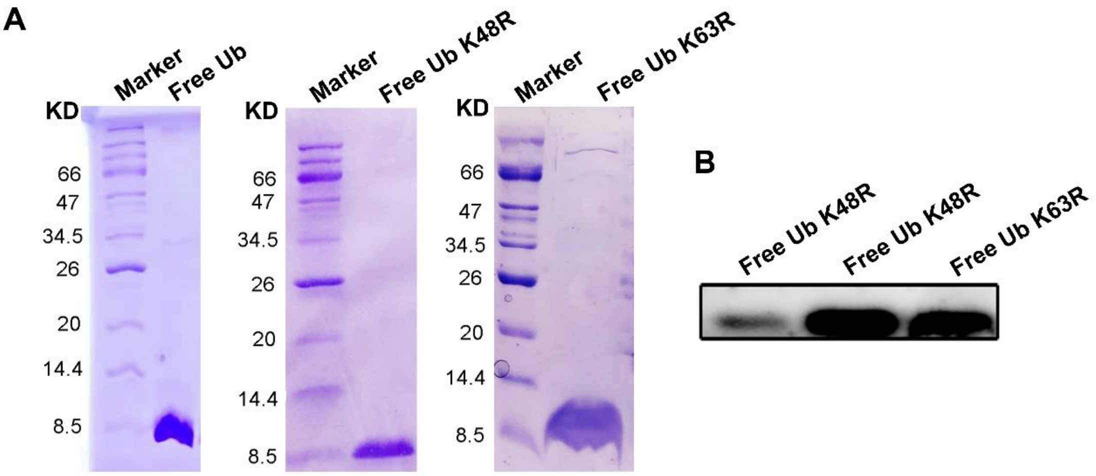

Figure S4. (A) Purified recombinant free ubiquitin and its mutants (K48R and K63R) were stained with Coomassie staining. The Coomassie staining was performed by Ubbiotech (Changchun, China). (B) Recombinant free ubiquitin and its mutants (K48R and K63R) were verified by Western blotting.

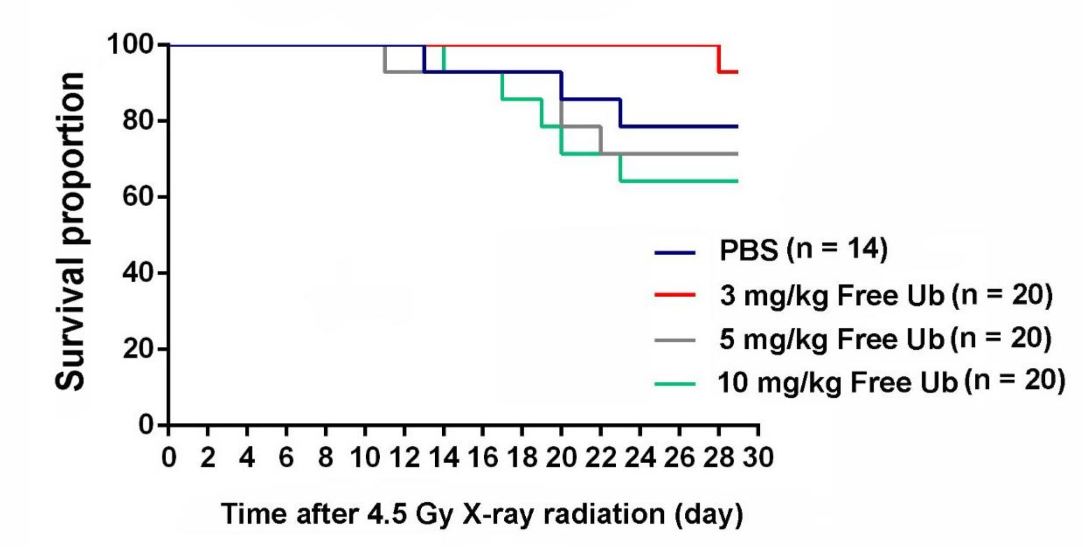

Figure S5. Kaplan-Meier survival analyses of C57BL/6J mice after 7 Gy TBI treated with PBS or indicated dose of free ubiquitin (Ub).

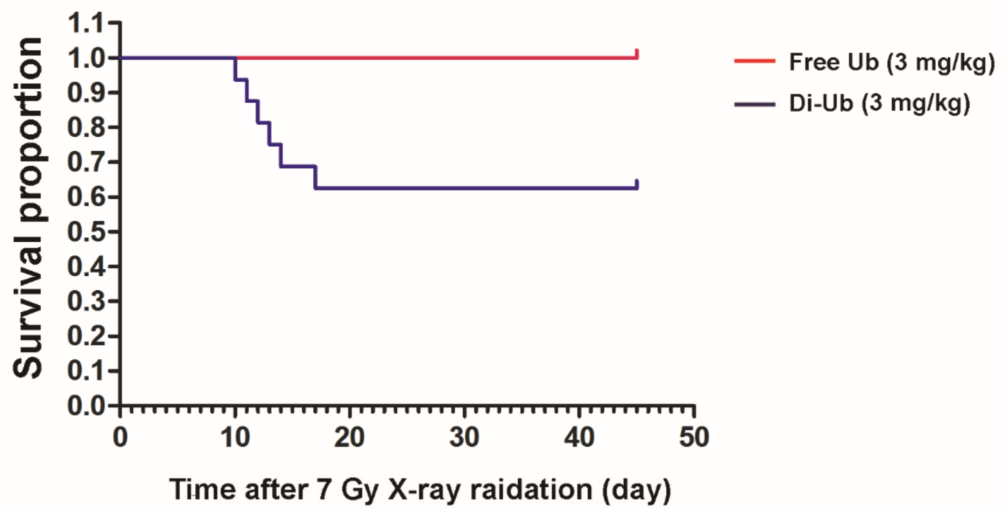

Figure S6. Kaplan-Meier survival curve of mice after 7 Gy TBI treated with free ubiquitin (Ub) or di-ubiquitin (Di-Ub).C57BL/6J mice were randomly divided into two groups (n = 20 for each group): 1) i.p. injection of free ubiquitin in a 100-μL volume PBS (3-10 mg free ubiquitin/kg animal body weight); 2) i.p. injection of Di-Ub in a 100-μL volume PBS (3-10 mg Di-Ub/kg animal body weight). Mice were exposed to 7 Gy X-ray radiation one hour after injection.

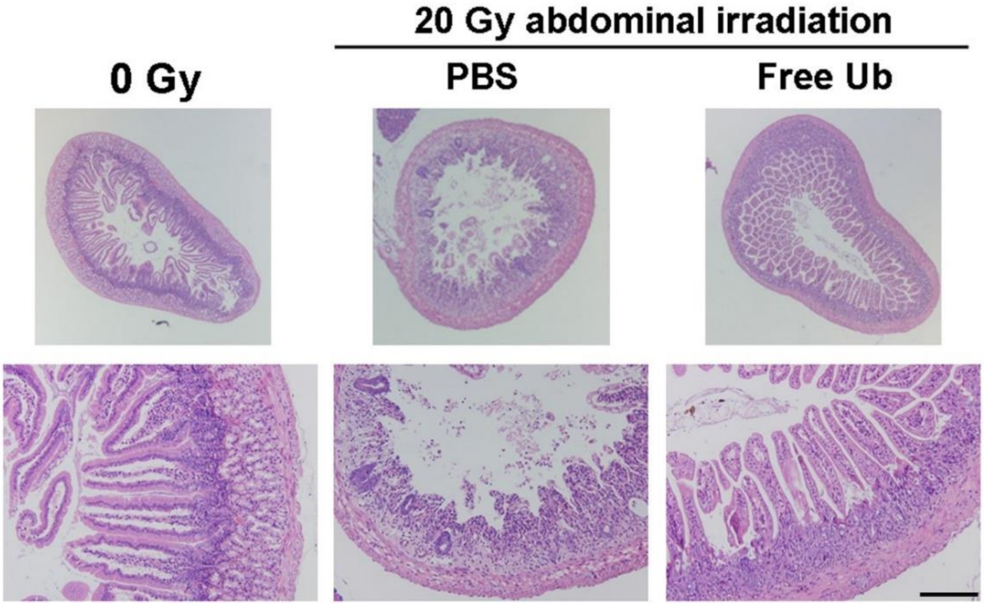

Figure S7. Representative H&E-stained sections of the small intestines. Mice were i.p. injected with PBS or free ubiquitin (Ub). Then, mice were exposed to 0 or 20 Gy abdominal irradiation. Small intestines form mice were harvested for pathological examination 3.5 days post radiation. Bar = 100 μm.

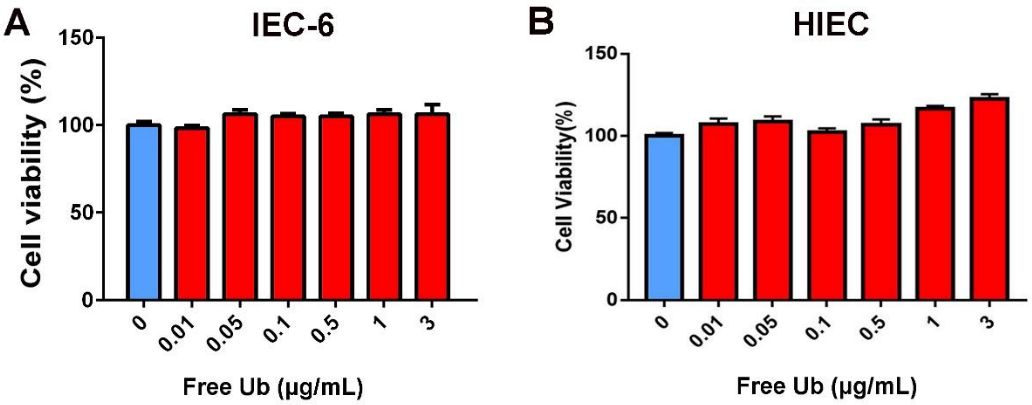

Figure S8. (A) IEC-6 and (B) HIEC cells were pretreated with indicated concentration of free ubiquitin. Cell viability was measured 72 h later by CCK-8 based assay.

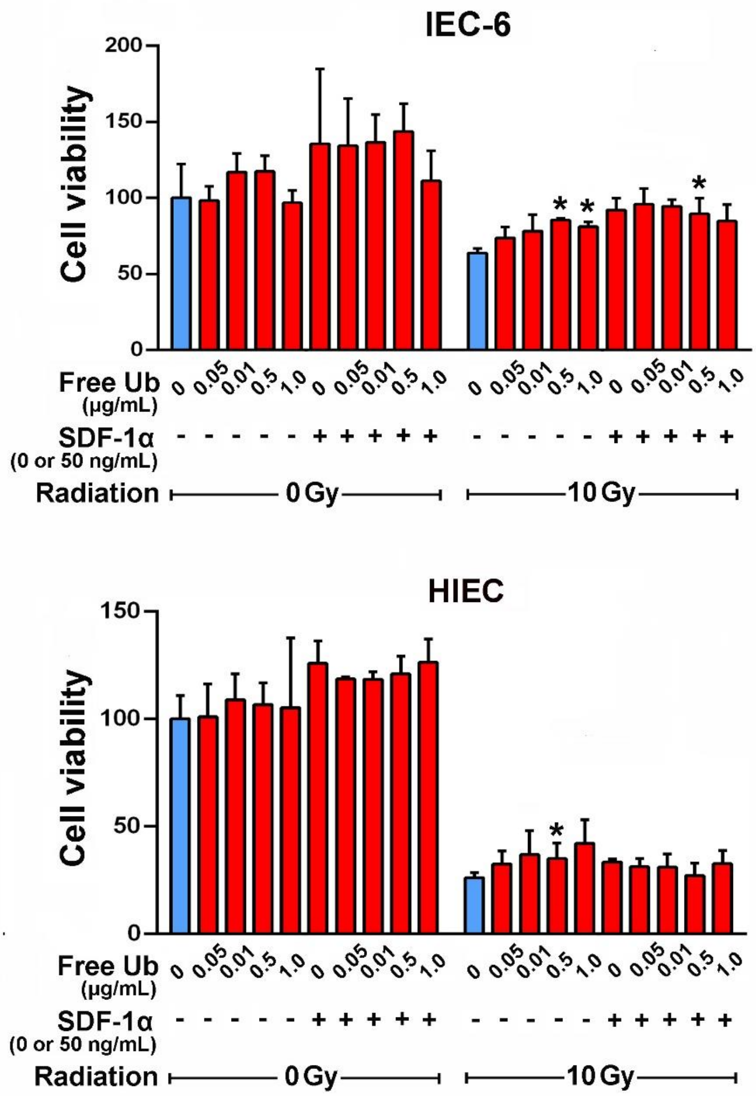

Figure S9. The effect of free Ub plus SDF-1α on the cell viability of IEC-6 and HIEC cells. Cells were pretreated with various concentration of SDF-1α or/and free Ub (0.5 μg/mL) for 8 h, followed by 10 Gy radiation. Cell viability was measured 72 h after radiation by CCK-8 based assay. **P* < 0.05; ** *P* < 0.01 compared with the control group.

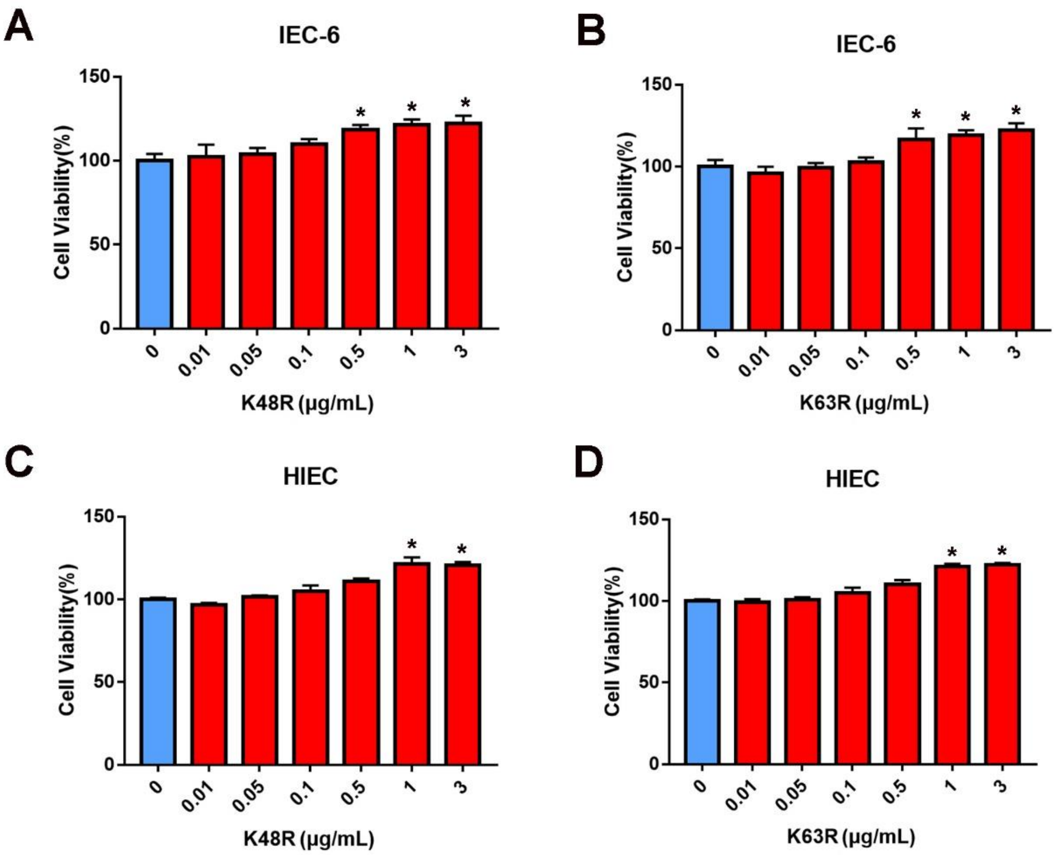

Figure S10. (A)-(D) IEC-6 and HIEC cells were pretreated with indicated concentration of free ubiquitin mutants (K48R or K63R). Cell viability was measured 72 h after 10 Gy radiation by CCK-8 based assay. **P* < 0.05 compared with the control group.

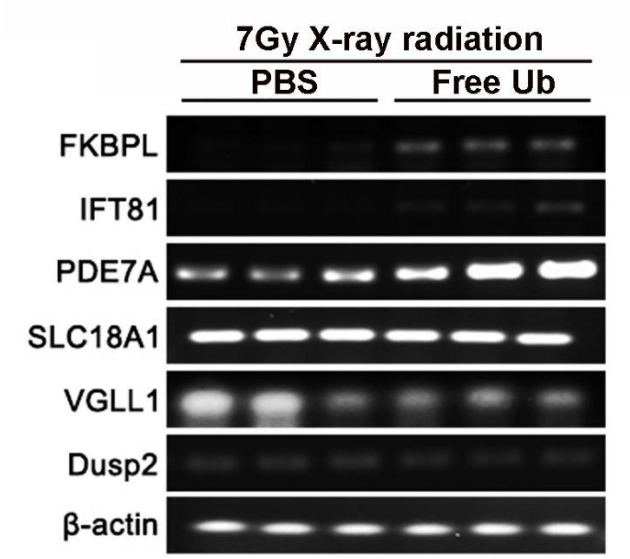

Figure S11. Mice were i.p. treated with PBS or free ubiquitin (UB) after 7 Gy radiation (n = 3). The expression of the seven genes of mouse intestines were analyzed by RT-PCR.

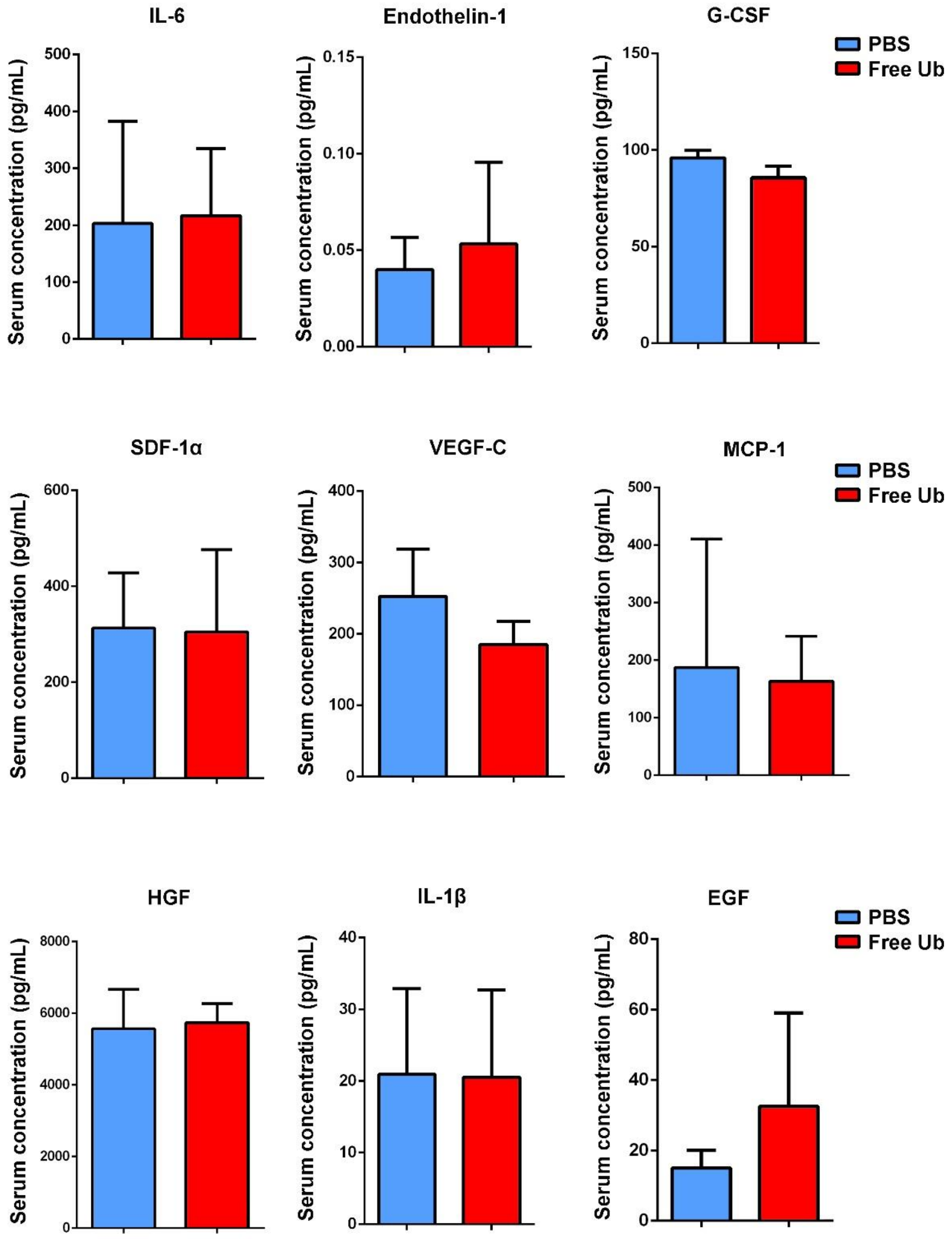

Figure S12. Mice were i.p. treated with PBS or free ubiquitin (Ub) before 7 Gy radiation. Serum concentration of the nine cytokines in mice was measured 5 days after radiation.

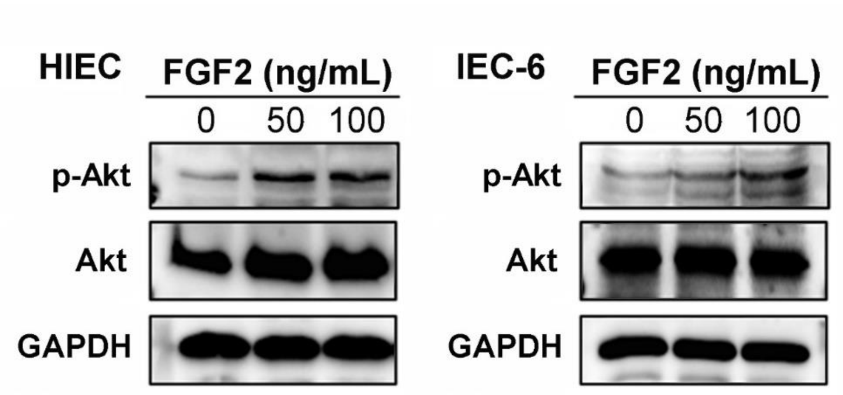

Figure S13. FGF2 promoted Akt phosphorylation. HIEC or IEC-6 cells were mock treated or treated with 50 and 100 ng/mL FGF2 for 20 min. Akt and p-Akt was analyzed by Western blotting.
